# Plant-pollinator trait matching affects pollen transfer but not feeding efficiency of Australian honeyeaters (Aves, Meliphagidae)

**DOI:** 10.1038/s42003-025-07693-w

**Published:** 2025-03-01

**Authors:** Amanda E. Hewes, Todd J. McWhorter, Alejandro Rico-Guevara

**Affiliations:** 1 Department of Biology, University of Washington, Seattle, WA USA; 2 Burke Museum of Natural History and Culture, Seattle, WA USA; 3 School of Animal & Veterinary Sciences, University of Adelaide, Roseworthy, SA Australia

**Keywords:** Behavioural ecology, Animal behaviour

## Abstract

Animal pollination is common among flowering plants. Increased morphological matching between floral and pollinator traits is thought to increase pollen transfer and feeding efficiency, but we lack studies that empirically demonstrate this. Working with Australian honeyeaters, we find that there is positive correlation between bill-corolla matching and pollen deposition at flowers, but no correlation with how efficiently birds can extract floral nectar. The species with the lowest bill-corolla matching deposited the fewest pollen grains but had the highest feeding efficiency, showing that bill-corolla matching expectations were met on the plant side of this interaction but not on the pollinator side. Finally, we find different interspecific patterns of pollen deposition at the scales of a single flower visit versus the landscape, due to differences in patterns of plant visitation. This work illustrates the need for more studies that directly correlate trait matching to fitness proxies of plants and avian pollinators.

## Abstract (web summary)

Experimental quantification of fitness proxies in bird-plant pollination interactions shows that trait matching correlates with pollen deposition on floral stigmas, but does not affect how efficiently birds extract nectar.

## Introduction

Animal pollination is a classic example of mutualism^1^; animal pollinators harvest rewards such as nectar or pollen, while plants receive pollination services that allow them to successfully reproduce. However, animal pollination commonly results in large amounts of pollen loss^2^. In response to selective pressures placed by effective pollinators, floral structures can evolve to be more restrictive (e.g., longer and/or narrower corolla) and can filter access to floral rewards to species that can successfully pollinate^3,4^. When floral morphology is more restrictive, nectar is accessed when a pollinator is feeding in a position that maximizes the likelihood of contact with floral reproductive structures^5–8^. This process reinforces morphological similarity between floral structures and pollinator feeding parts, resulting in plant-pollinator trait matching^3,4^. Trait matching is known to be important in structuring pollination interactions in numerous insect and avian pollination systems^3,9–17^ and plant traits that are involved in trait matching tend to have strong signatures of pollinator-mediated selection^18^. Depending on the strength and spatial and temporal variability in pollination selection, trait matching can be the result of, and reinforce, reciprocal coevolution between plants and pollinators^19–21^.

In avian pollination systems, hummingbirds in the Neotropics consistently show trait matching with flowers they visit^22–35^ and growing evidence suggests that sunbirds in Africa do as well^36,37^. While this pattern has been observed repeatedly in nature, experimental evidence that supports the mechanistic steps by which bill-corolla matching can positively contribute to fitness for both parties, measured via proxies such as feeding efficiency and pollen transfer efficiency, is lacking^34^. Studies that experimentally measure per-visit pollen removal and deposition in avian pollination systems are largely restricted to hummingbirds and these studies have found a positive correlation between bill-corolla matching and pollen transfer success^17,38–41^. However, none of these studies have simultaneously measured feeding efficiency (nectar volume consumed/second). Conversely, many studies have examined how floral traits affect bird nectar extraction but have done so using artificial “flowers” (i.e., plastic tubing) that vary morphologically in controlled ways (e.g., length or curvature)^42–45^. The few studies of bird feeding rates at real flowers did not measure how much pollen was removed or deposited^24,26,27,46^. We therefore lack studies that simultaneously measure the correlation between bill-corolla matching and fitness proxies for both interaction partners.

Beyond studying pollen removal and transfer in a single flower visit^17,38,39,47^, it is important to know the extent to which interspecific patterns in pollen transfer efficiency across bird species will remain consistent when scaling up to landscape-level foraging. Some studies have measured the effect of floral morphology and bill-corolla matching on hummingbird contributions to seed set in wild plants^48^, but it is unknown whether interspecific differences are due to differences in pollen transfer efficiency at the level of each flower visit or differences in the number of visits. Spatiotemporal variation in pollinator-mediated selection is likely to be an important determinant of selection on plant morphology^49^, as outlined in the geographic mosaic theory of plant-pollinator coevolution^21^, so to understand the true evolutionary relevance of bill-corolla matching one would ideally measure its effect at the scale of the flower visit, and then determine whether those effects scale across a visit to an entire plant or multiple plants across the landscape.

Studies on the prevalence and importance of trait matching in pollination interactions are most common in hummingbirds pollination systems (but see refs. ^36,37,50,51^). Examining the role of bill-corolla matching in a system with less specialized bird-plant interactions can provide a broader understanding of these relationships. Honeyeaters, the most common avian pollinators in Australasia^50,52^, have more generalized interactions with plants than do hummingbirds^53–55^ and are therefore an ideal study system to fill this knowledge gap. While there have been studies examining honeyeater pollination services to native plants^50,56–60^, the role of bill-corolla matching in shaping interspecific differences in pollen transfer services has been minimally investigated^50,51^ and no study has simultaneously considered fitness proxies for both interaction partners. In this study we examined the role of trait matching, specifically bill-corolla matching, in determining interspecific differences in honeyeater pollen transfer and feeding efficiency at the Australian shrub, *Eremophila maculata* (Scrophulariaceae, spotted emu bush), in the temperate mallee of South Australia. We studied three honeyeater species that constituted >90% of all visits to *E. maculata* in the field. We used high-speed videography of wild-caught birds feeding at *E. maculata* flowers in captivity to simultaneously calculate feeding and pollen transfer efficiency in a controlled, yet real-to-life way. We then combined the laboratory data on pollen deposition with field recordings of honeyeater visitation to *E. maculata* to investigate whether the interspecific differences in pollen deposition at a single flower hold across the landscape. We aimed to answer three questions: (1) Do honeyeater species vary in feeding and pollen transfer efficiency at *Eremophila maculata* flowers?, (2) Can that interspecific variation be explained by variation in bill-corolla matching?, and (3) Do interspecific patterns of pollen deposition hold across increasing spatial scales? We predicted there would be a positive correlation between bill-corolla matching and pollen removal, pollen deposition, and feeding efficiency across honeyeater species. Scaling up to the landscape level, we hypothesize that there will likely be interspecific differences in pollen deposition across spatial scales due to interspecific differences in plant visitation. We focus on pollen deposition for this across-scale extrapolation because it is the most likely limiting factor for successful pollination among the variables we measured.

## Results

### Pollen transfer across honeyeater species

**Fig. 1:**
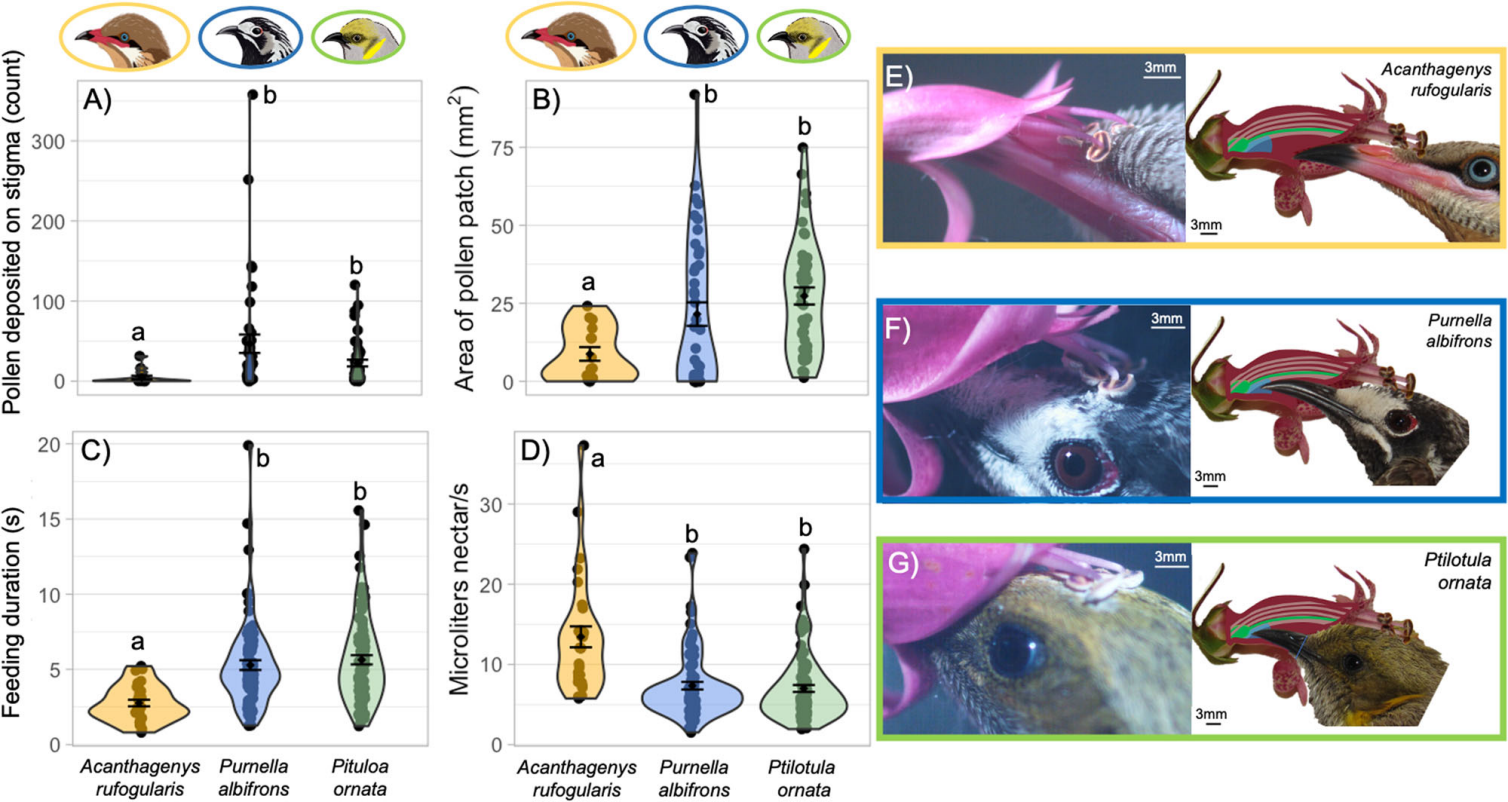
Patterns in pollen transfer and feeding efficiency across honeyeaters. Differences measured in pollen transfer efficiency (**A**, **B**) and feeding efficiency (**C**, **D**) across honeyeater species. *A. rufogularis* deposited (**A**) and removed (**B**) significantly less pollen than *Pu. albifrons* or *Pi. ornata*, but the latter two species were not significantly different. *A. rufogularis* fed faster (**C**) and therefore had higher volumetric uptake rate (**D**) than *Pu. albifrons* or *Pi. ornata*, but the latter two species were not significantly different. **E**–**G** Left: still images from high-speed videos showing pollen deposition on each species, Right: bird species (to scale) overlaid on cross sections of *Eremophila maculata* flowers, illustrating differences in bill insertion depth, which in turn affects pollen deposition location and the distance the tongue must travel to access the nectar pool; these differences played a role in determining the interspecific differences illustrated in (**A**–**D**). While *A. rufogularis* has a greater bill tip-to-nectar distance than *Pu. albifrons* or *Pi. ornata*, it also has a larger tongue and can therefore likely capture more nectar per lick (see “Discussion”). In (**A**–**D**) the mean of each sample is indicated with a diamond and the error bars show $\pm \,$1 standard error. Letters over each violin plot show which species are significantly different. Results from modeling are shown in Table 1 and summary statistics are shown in Table S1. Bird illustrations above panels A & B are by Kindall Murie (to scale).

We examined three components of pollen transfer efficiency: (1) the number of pollen grains deposited on the stigma; (2) the duration of contact between the bird’s body and the anthers; and (3) the size of the pollen patch on the bird resulting from contact with anthers. We found significant interspecific differences in the number of pollen grains deposited on stigmas (*p* = 0.004). *A. rufogularis* individuals deposited fewer pollen grains on stigmas than individuals of *Pi. ornata* (Cohen’s *d* = 0.86, *p* = 0. 017) or *Pu. albifrons* (Cohen’s *d* = 0.82, *p* = 0. 0013). The number of pollen grains deposited did not significantly differ between *Pu. albifrons* and *Pi. ornata* (Cohen’s *d* = 0.44, *p* = 0.46) (Fig. 1A, Table 1, S1).

**Table 1:** Interspecific differences in pollen transfer and feeding efficiency across honeyeaters

Model	Response	Fixed effects			*P* value		Effect size (Cohen’s *d*)	Marginal R^2^ (trigamma)	Conditional R^2^ (trigamma)
		Effect *Pairwise levels*	Raw regression coefficient (link scale)	Transformed regression coefficient (response scale)	Fixed effect	Pairwise levels			
1	Number of pollen grains deposited on stigma	Species *Ar–Pa* *Ar–Po* *Pa–Po*	−1.68	0.187	**0.004**	**0.0027**	0.82	0.11	0.19
			−1.27	0.281		**0.031**	0.86		
			0.407	1.5		0.38	0.44		
2	Anther contact duration (s)	Species *Ar–Pa* *Ar–Po* *Pa–Po*	−0.0615	0.94	**0.0045**	0.97	0.15	0.14	0.27
			−0.584	0.558		0.054	0.94		
			−0.523	0.593		**0.011**	0.98		
3	Area of pollen patch (mm2)	Species *Ar–Pa* *Ar–Po* *Pa–Po*	−1.18	0.306	**0.00057**	**0.0005**	0.71	0.25	0.36
			−1.11	0.331		**0.0008**	1.3		
			0.0783	1.08		0.903	0.27		
4	Feeding duration (s)	Species *Ar–Pa* *Ar–Po* *Pa–Po*	−0.634	0.53	**0.0019**	**0.0050**	1.1	0.18	0.40
			−0.683	0.505		**0.0018**	1.3		
			−0.0488	0.952		0.94	0.12		
5	Feeding efficiency (µL/s)	Species *Ar–Pa* *Ar–Po* *Pa–Po*	0.601	1.82	**0.0018**	**0.0075**	1.04	0.17	0.37
			0.691	1.99		**0.0013**	1.1		
			0.0898	1.09		0.81	0.084		

Outputs of models examining interspecific differences in pollen transfer and feeding metrics across honeyeater species. Regression coefficients are provided as raw values (on the scale of the link function) and exponentiated to be on the response scale. Raw regression coefficients can be interpreted as the expected change in the ln(counts) for every 1 unit increase in X. Transformed regression coefficients can be interpreted as a multiplicative factor by which the mean of Y changes for every 1 unit increase in X. Significant *p* values are bolded.

*Ar* Acanthagenys rufogularis, *Pa* Purnella albifrons, *Po* Ptilotula ornata.

There were significant interspecific differences in the duration of anther contact (*p* = 0.0045) and pollen patch area (*p* = 0.00057). For duration of anther contact, *Pu. albifrons* had the shortest contact duration, followed by *A. rufogularis*, with *Pi. ornata* having the longest (Table S1)*. Pu. albifrons* and *Pi. ornata* were different from each other (Cohen’s *d* = 0.98, *p* = 0. 011), but neither differed significantly from *A. rufogularis* (*Pi. ornata—*Cohen’s *d* = 0.94, *p* = 0.054; *Pu. albifrons—*Cohen’s *d* = 0.15, *p* = 0.97) (Table 1). For area of the pollen patch, *A. rufogularis* individuals had a smaller area of pollen patch than individuals of *Pi. ornata* (Cohen’s *d* = 1.3, *p* = 0.0008) or *Pu. albifrons* (Cohen’s *d* = 0.71, *p* = 0.0005), but *Pu. albifrons* and *Pi. ornata* did not significantly differ (Cohen’s *d* = 0.27, *p* = 0.903) (Fig. 1B, Table 1, S1).

### Feeding efficiency across honeyeater species

Feeding duration was measured directly from high-speed videos (see “Methods”). Feeding efficiency was calculated as the microliters of nectar consumed, which was standardized to 30microliters across all trials, divided by feeding duration (see “Methods”). There were significant interspecific differences in feeding duration (*p* = 0.0019) and efficiency (*p* = 0.0018; Fig. 1C, D, Table 1, S1). *A. rufogularis* depleted the nectar faster and as a result had a higher feeding efficiency (Fig. 1C, D, Table S1), than *Pi. ornata* (feeding duration—Cohen’s *d* = 1.3, *p* = 0.0018; feeding efficiency—Cohen’s *d* = 1.1, *p* = 0.0013) and *Pu. albifrons* (feeding duration—Cohen’s *d* = 1.1, *p* = 0.005; feeding efficiency—Cohen’s *d* = 1.04, *p* = 0.0075) (Fig. 1C, D, Table 1)*. Pu. albifrons* and *Pi. ornata* were not significantly different in either feeding metric (feeding duration—Cohen’s *d* = 0.12, *p* = 0.94; feeding efficiency—Cohen’s *d* = 0.084, *p* = 0.81) (Fig. 1C, D, Table 1).

### Bill-corolla matching between honeyeaters and *Eremophila maculata*

**Fig. 2:**
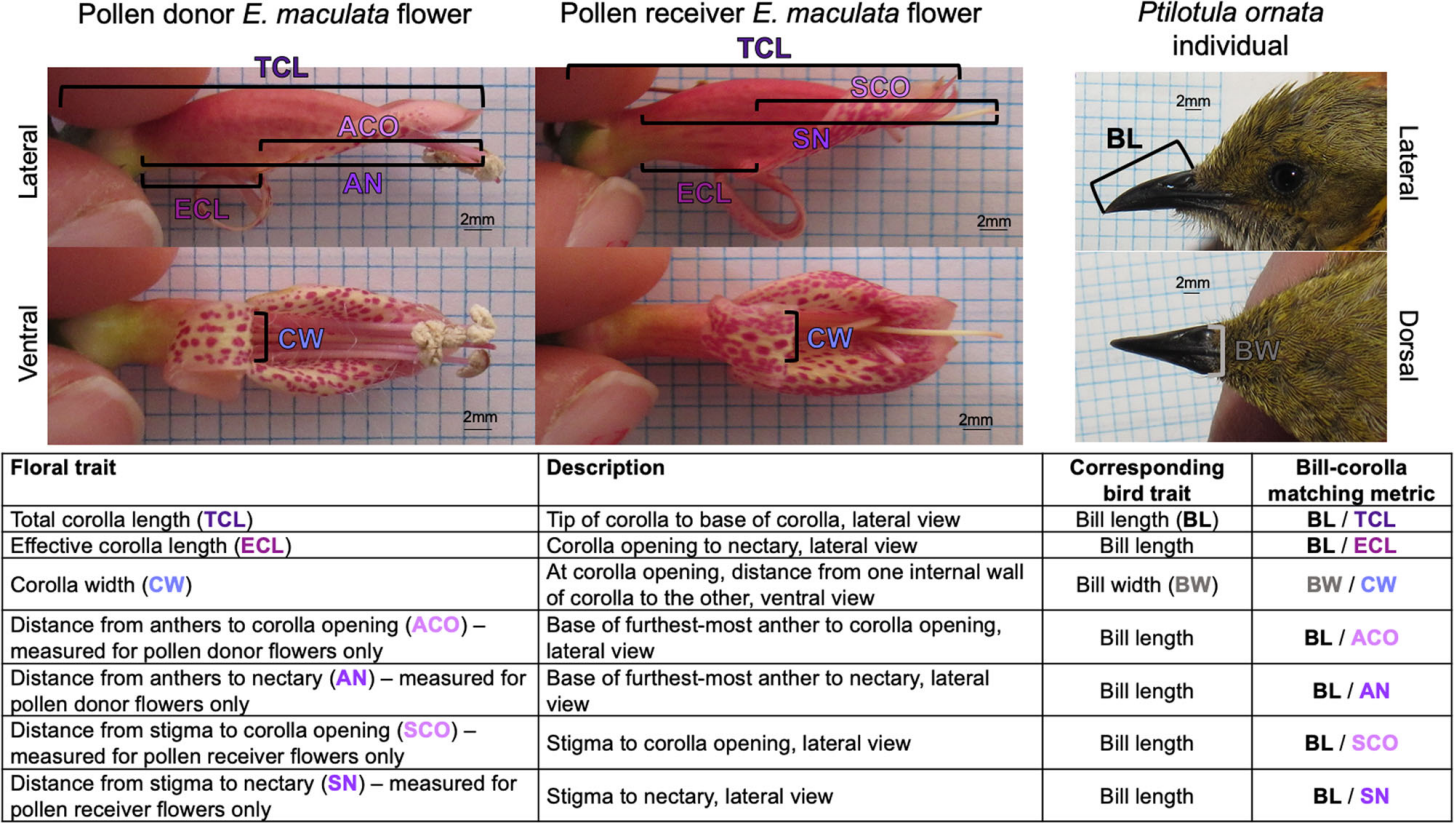
Quantifying bill-corolla matching. Floral and bird morphometrics taken to quantify bill-corolla matching. There are no expectations for a particular number that indicates best matching between bill and flower, for example a bill length to total corolla length (BL/TCL) ratio of 1, because the matching is an emergent property of the bill-flower interaction and not all bill-corolla matching metrics can simultaneously equal the same value.

**Fig. 3:**
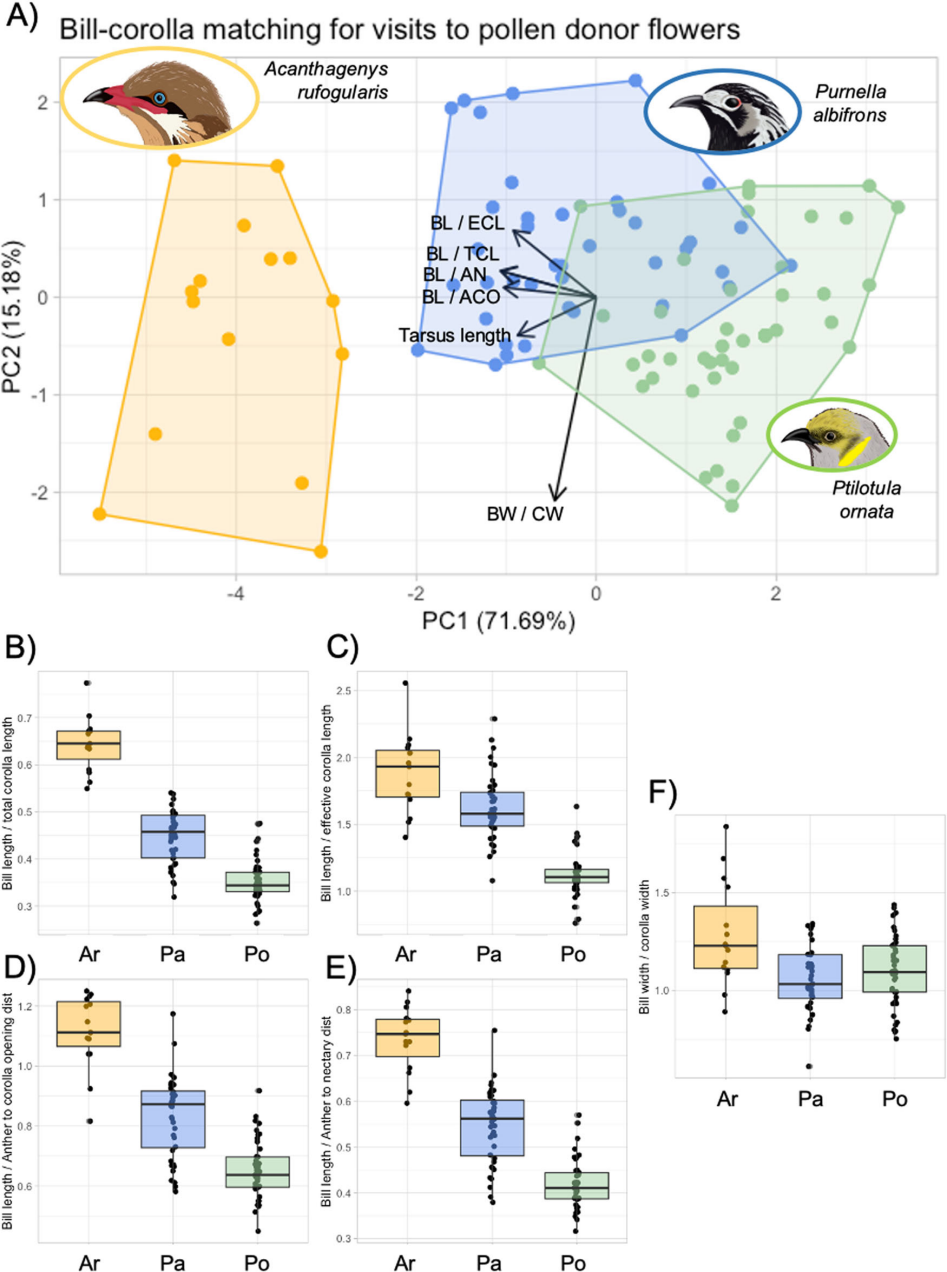
PCA and raw data of bill-corolla matching metrics. **A** PCA of bill-corolla matching metrics measured from honeyeater visits to pollen donor *Eremophila maculata* flowers. The loadings of each bill-corolla matching variable are illustrated with arrows. **B**–**F** Raw data of bill-corolla matching metrics showing the same trends visualized in the PCA. In (**A**) abbreviations are as follows: BL/TCL = bill length/total corolla length, BL/ECL = bill length/effective corolla length, BW/CW = bill width/corolla width, BL/ACO = bill length/distance from anthers to corolla opening, BL/AN = bill length/distance from anthers to nectary, BL/SCO = bill length/distance from stigma to corolla opening BL/SN = bill length/distance from stigma to nectary. See Table S2 for PCA loadings and Fig. 2 for descriptions of all bill-corolla matching variables. See Fig. S1 for PCA of bill-corolla matching metrics measured from honeyeater visits to pollen receiver flowers. Bird illustrations (to scale) are by Kindall Murie.

The crux of this study was to investigate whether variability in bill-corolla matching metrics is related to differences in feeding and pollen transfer efficiency across honeyeater species. We quantified bill-corolla matching using several ratios of bill and corolla linear morphometrics (Fig. 2). We then used PCA to create a multivariate measure of trait matching that uses all of the ratios measured and handles the inherent correlation between them (Fig. 2). We ran one PCA for bird interactions with pollen donor flowers (flowers with anthers intact, see “Methods” and Fig. 3A) and a second PCA for bird interactions with pollen receiving flowers (flowers with anthers removed and stigma only, see “Methods” and Fig S1A) and we distinguish between these PC scores by noting “pollen donor PC1” and “pollen receiver PC1”, “pollen donor PC2” and “pollen receiver PC2”, etc. The three honeyeater species were clearly distinguished by PC scores (Fig. 3A), and the PCAs for pollen donor and pollen receiver interactions were very similar (compare Fig. 3A and Fig. S1A). For the pollen donor PCA, PC1 explained 71.7% of the variation and PC2 explained 15.2% (Fig. 3A). For the pollen receiver PCA, PC1 explained 70.7% of the variation and PC2 explained 16.4% (Fig. S1A). The loadings of the bill-corolla matching variables were the same for both pollen donor and pollen receiver PCAs (Table S2). In both PCAs, PC1 was dominated by bill-corolla matching length variables and tarsus length, while PC2 was dominated by bill-corolla width matching (Table S2).

**Fig. 4:**
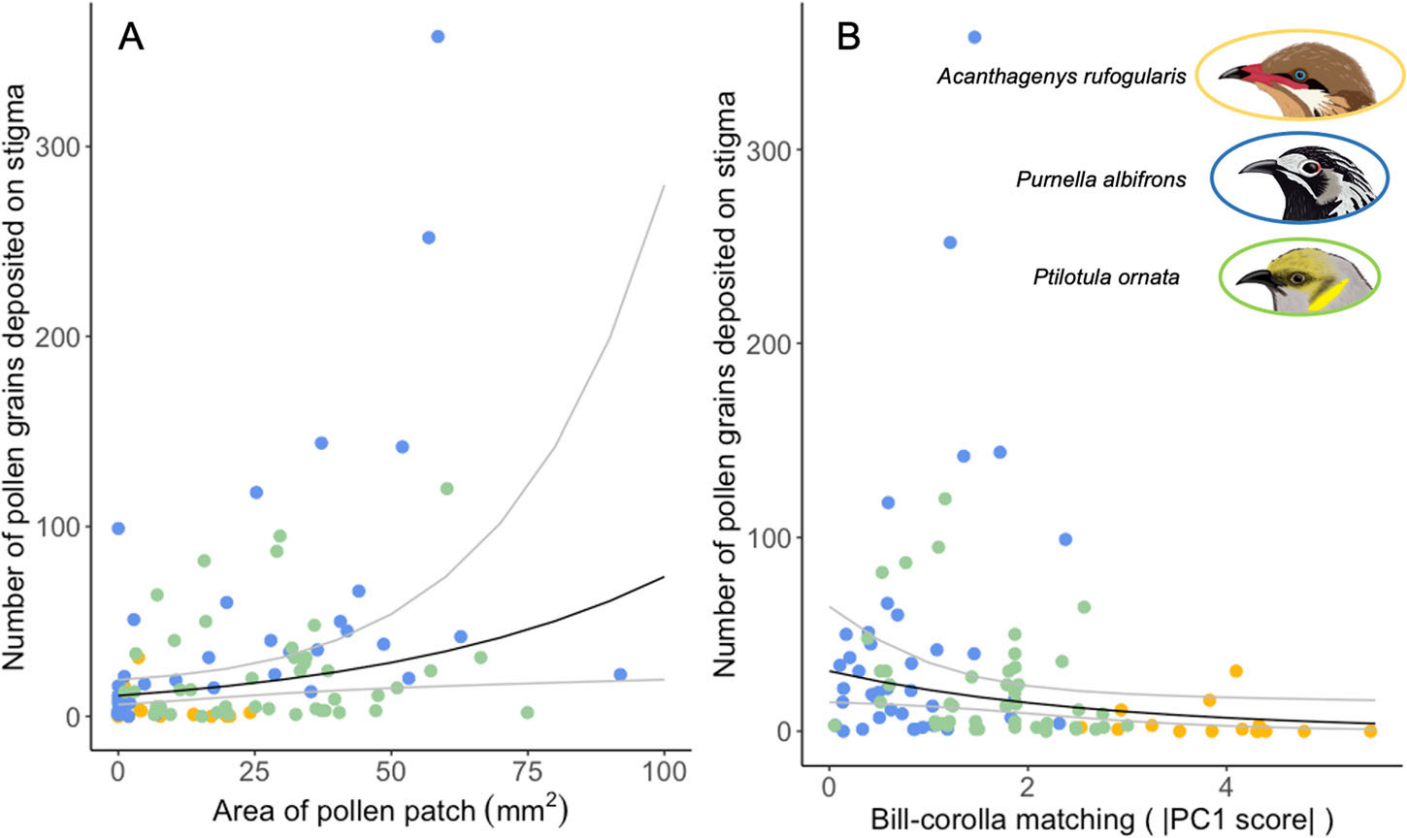
Factors that best explain pollen deposition of honeyeaters at *Eremophila maculata.* Predictors that best explain the number of pollen grains deposited on *Eremophila maculata* stigmas: area of the pollen patch (**A**) and pollen receiver |PC1 score| (**B**). The area of the pollen patch is positively correlated with pollen deposition, while |PC1 score| is negatively correlated with pollen deposition, meaning that has the bill-corolla matching worsens (primarily in length) the amount of pollen deposited is reduced. See Table 2 for full statistical results. Solid black lines are predictions made with the ggpredict() function while holding one predictor constant at its mean value. Gray lines show 95% confidence intervals around those predictions. Bird illustrations (to scale) are by Kindall Murie.

*A. rufogularis* had the lowest PC scores of all three species (Fig. 3A), meaning that *A. rufogularis* individuals have the largest bills relative to the size of *E. maculata* flowers out of all three species; this is confirmed when looking at the raw data of ratios between bill and corolla morphometrics (Fig. 3B–F). *A. rufogularis* bills are largest relative to *E. maculata* flowers in metrics of length (Fig. 3B–E) and width (Fig. 3F). Conversely, *Pi. ornata* had the highest PC scores of all three species (Fig. 3A), meaning that *Pi. ornata* individuals had the smallest bills relative to the size of *E. maculata* flowers out of all three species (Fig. 3A). The raw data of ratios between bill and corolla morphometrics shows that *Pi. ornata* bills are smallest relative to *E. maculata* flowers in metrics of length (Fig. 3B–E) but are a similar size to *E. maculata* flowers width (Fig. 3F). Finally, *Pu. albifrons* had scores that were intermediate between those of *A. rufogularis* and *Pi. ornata* (Fig. 3A). In some trials the bill of *Pu. albifrons* individuals was larger than the *E. maculata* flower it fed at, while in other trials the bill was smaller than the flower. This is once again seen in the raw data, where *Pu. albifrons* has intermediate values for all metrics of length (Fig. 3B–E) and a similar size to *E. maculata* flowers in width (Fig. 3F). In the models below where bill-corolla matching is investigated (Fig. 4, Table 2), PC scores were converted to absolute value for better interpretability and model fitting. Zero is the mean of each principal component and deviation from zero indicates a larger or smaller bill relative to *E. maculata* flowers (Fig. 3A).

**Table 2:** Correlations between bill-corolla matching and pollen transfer and feeding efficiency

Model	Response	Fixed effects			*P* value	Marginal R^2^ (trigamma)	Conditional R^2^ (trigamma)
		Effect	Raw regression coefficient (link scale)	Transformed regression coefficient (response scale)			
6	Area of pollen patch (mm2)	|PC1|	0.031	1.03	0.84	0.0089	0.55
		|PC2|	−0.16	0.85	0.36		
7	Number of pollen grains deposited on stigma	Area of pollen patch	0.019	1.02	**0.019**	0.15	0.39
		|PC1|	−0.37	0.69	**0.033**		
		|PC2|	−0.18	0.84	0.49		
8	Feeding efficiency (µL/s)	|PC1|	0.043	1.04	0.48	0.0083	0.35
		|PC2|	−0.015	0.99	0.81		

Outputs of models examining correlation between bill-corolla matching and pollen transfer and feeding efficiency. Regression coefficients are provided as raw values (on the scale of the link function) and exponentiated to be on the response scale. Raw regression coefficients can be interpreted as the expected change in the ln(counts) for every 1 unit increase in X. Transformed regression coefficients can be interpreted as a multiplicative factor by which the mean of Y changes for every 1 unit increase in X. Significant *p* values are bolded.

*Ar* Acanthagenys rufogularis, *Pa* Purnella albifrons, *Po* Ptilotula ornata.

### Relationship between bill-corolla matching and pollen transfer and feeding efficiency

There was no significant correlation between area of the pollen patch and bill-corolla matching (Table 2). The marginal R2 (fixed effects only) of the model was very low at 0.0089, showing that bill-corolla matching explains almost none of the interspecific variation in area of the pollen patch. The conditional R2 of the same model, which considers the fixed effects and the nested random effects of individual within species, was 0.55, which is a substantial increase from the marginal R2 and shows that much of the variation is explained by the nested random effects of individual within species.

There was a significant correlation between pollen deposition on stigmas and bill-corolla matching (Fig. 4, Table 2). The number of pollen grains deposited on *E. maculata* stigmas was negatively correlated with the absolute value of the bill-corolla matching PC1 score ($\beta$ = −0.37, *p* = 0.033), with a one unit increase in PC1 score resulting in a 31% decrease in mean pollen deposition (Fig. 4, Table 2). The bill-corolla matching PC2 score had a slight negative, but non-significant relationship with pollen deposition ($\beta$ = −0.18, *p* = 0.49). Pollen deposition was also positively correlated with the area of the pollen patch picked up from the previous flower visit ($\beta$ = 0.019, *p* = 0.019), with a one unit increase in pollen patch area resulting in a 2% increase in mean pollen deposition (Fig. 4, Table 2). The marginal R2 of the model was 0.15, while the conditional R2 was 0.39 (Table 2).

**Fig. 5:**
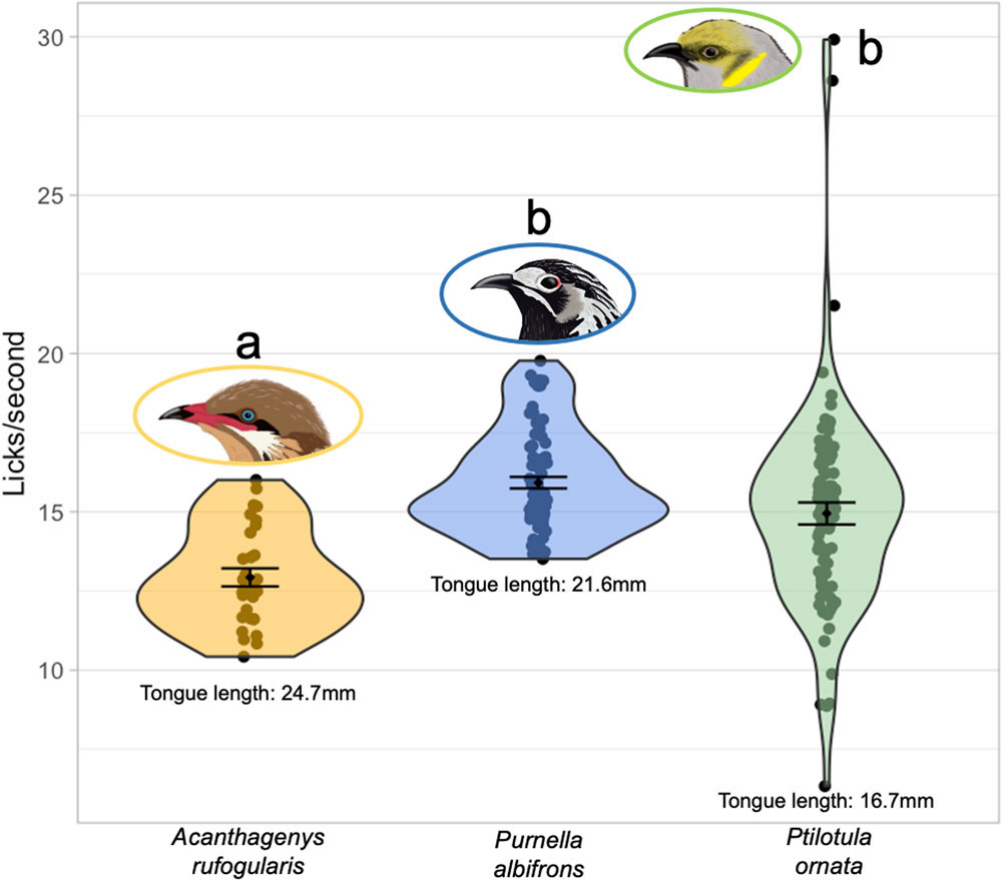
Differences in licking rate across honeyeaters. Licking rates of *A. rufogularis*, *Pu. albifrons* and *Pi. ornata* at *Eremophila maculata* flowers measured using high-speed videography. Letters denote which species are significantly different. Tongue lengths are provided for reference from ref. ^62^. Bird illustrations (to scale) are by Kindall Murie.

When examining the correlation with bill-corolla matching, we focused on volumetric uptake rate only as it had the same interspecific differences as total time spent feeding so examining both would be redundant. There was no correlation between feeding efficiency and bill-corolla matching (Table 2). The marginal R2 of the model was 0.0083 while the conditional R2 was 0.35, showing that much of the variation is explained by the nested random effects of individual within species. We did not find a correlation between feeding efficiency and bill-corolla matching (Table 2) but there were interspecific differences (Fig. 1D, Table 1). To investigate possible reasons for these differences, we characterized licking rate and found that *A. rufogularis* had the lowest licking rate of the three species, despite having the highest feeding efficiency, while *Pu. albifrons* and *Pi. ornata* had similar licking rates that were higher than those of *A. rufogularis* (Fig. 5, Table S1).

### Scaling up to the pollination landscape

**Fig. 6:**
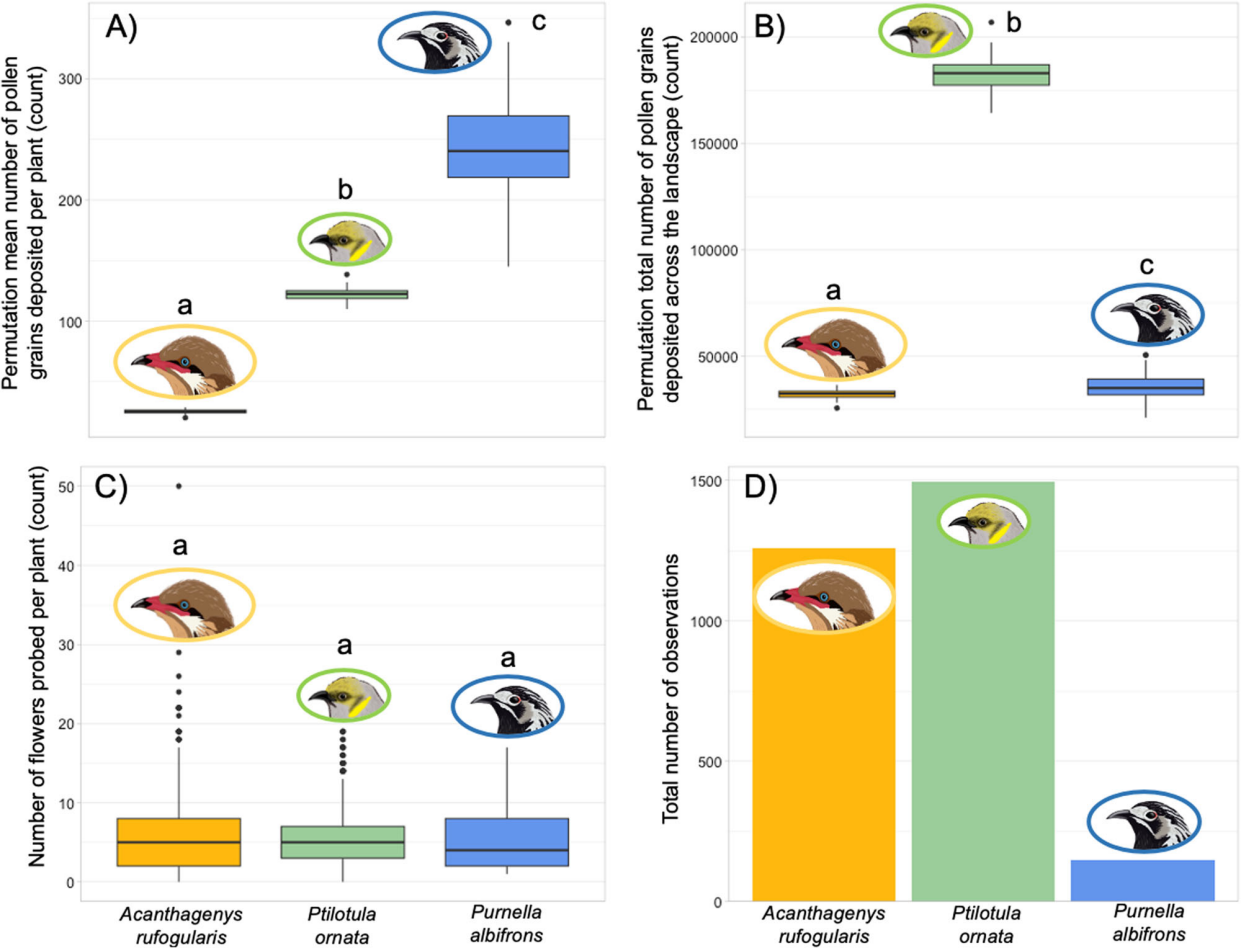
Differences in plant visitation and estimated landscape-scale pollen deposition across honeyeaters. Estimates of (**A**) mean pollen deposition per species during a visit to a single *Eremophila maculata* plant and (**B**) total pollen deposition across all *Eremophila maculata* plants that were monitored with camera traps. Differences cannot be explained by the number of flowers probed per plant by each species (**C**) but is largely due to a combination of differences in the total number of visits to *Eremophila maculata* plants (**D**) and difference in the ability to deposit pollen (Fig. 1A). In plots A and B each data point is the mean estimated pollen deposition from a single permutation run, and the distribution shown for each species is the mean estimated pollen deposition from all 100 permutation runs. Letters above boxplots denote which species are significantly different. Results from permutations and statistical analysis are shown in Table 3. Bird illustrations (to scale) are by Kindall Murie.

To determine whether interspecific patterns of pollen deposition from experiments held when considering patterns of foraging behavior, we combined the experimentally derived data on per-flower pollen deposition with camera trap data that quantified the number of flowers probed per visit and the total number of recorded visits. We estimated the per-plant number of pollen grains deposited by each species and the number of pollen grains deposited across the landscape. We found significant interspecific differences in estimated mean pollen deposition per plant visit and in total pollen deposition across the landscape (Fig. 6A, B, Table 3). *Pu. albifrons* had the highest estimated mean pollen deposition per plant visit, followed by *Pi. ornata* and *A. rufogularis* and the effect sizes across all pairwise comparisons of species were large (Fig. 6A, Table 3). The pattern was different at the landscape scale, where *Pi. ornata* had the highest estimated total pollen deposition, followed by *Pu. albifrons* and *A. rufogularis* (Fig. 6B, Table 3). While the pattern differed across scales, the effect sizes across all pairwise comparisons of species were still large at the landscape scale (Fig. 6B, Table 3). There was no significant difference across species in the number of flowers probed per visit (Fig. 6C, Table 3), but *Pi. ornata* had the greatest number of visits recorded, followed by *A. rufogularis*, and *Pu. albifrons* (Fig. 6D, Table 3).

**Table 3:** Interspecific differences in estimated pollen deposition across the landscape

Species	Number of visits	Number of flowers probed per visit			Permutation mean number of pollen grains deposited per plant (count)			Permutation total number of pollen grains deposited across the landscape (count)		
*Acanthagenys rufogularis*	1258	5.6 $\pm \,$0.12			25.6 $\pm \,$0.163			32210 $\pm \,$205		
*Ptilotula ornata*	1495	5.4 $\pm \,$0.092			122 $\pm \,$0.463			182570 $\pm \,$692		
*Purnella albifrons*	146	5.2 $\pm \,$0.31			245 $\pm \,$3.98			35804 $\pm \,$581		

Species pair		*ANOVA* *F* value, *p* value	*Tukey* Estimate, *p* value	*Cohen’s d*	*ANOVA* *F* value, *p* value	*Tukey* Difference, *p* value	*Cohen’s d*	*ANOVA* *F* value, *p* value	*Tukey* Difference, *p* value	*Cohen’s d*
*Ar–Po*		1.94, 0.144	0.260, 0.19	0.0654	3335, **<2e-16**	−95.6, **<0.0001**	−26.6	27910, **<2e-16**	−148889, **<0.0001**	−28.2
*Ar–Pa*			0.458, 0.38	0.113		−218, **<0.0001**	−9.51		−2893, **0.0002**	−0.781
*Po–Pa––*			0.198, 0.83	0.0541		−122, **<0.0001**	−5.28		145996, **<0.0001**	24.2

Summary statistics and results from statistical analysis of pollen deposition at multiple scales. Summary statistics shown as mean $\pm \,$1 SE (except for number of visits). Number of visits and flowers probed per plant are raw data from camera traps. Pollen deposited per plant and pollen deposited across landscape are estimates from the permutation analysis. Significant *p* values are in bold.

*Ar* Acanthagenys rufogularis, *Pa* Purnella albifrons, *Po* Ptilotula ornata.

## Discussion

In this study we found that honeyeaters differed in their pollen removal, pollen deposition, and feeding efficiency at *E. maculata* flowers. While bill-corolla matching was correlated with pollen deposition on *E. maculata* stigmas, it was not correlated with pollen removal from flowers or with honeyeater feeding efficiency. The presence of a correlation between bill-corolla matching, particularly in bill length relative to corolla length, and pollen deposition on the stigma (Fig. 4B) is not unexpected due to the floral morphology of *E. maculata*, where the style and stigma are oriented in the longitudinal plane of the flower (Fig. 2). During a given visit, the length of the flower relative to the bill plays a large role in determining where the stigma will make contact with the bird’s head (Fig. 1E–G). Interspecific differences in pollen patch area are not explained by bill-corolla matching (Table 2), but the location of pollen deposition on the birds’ body could have implications for the persistence of that pollen post-anther contact (Fig. 1E-G). In all *A. rufogularis* visits the pollen was deposited on the base of the bill, lores (feathers between gape and eye), and/or lower forehead feathers (immediately posterior to the bill; Fig. 1E). This matches the findings in the morphological PCA (Fig. 3A), which demonstrated that all *A. rufogularis* individuals have bills larger, specifically longer and wider, than the flowers they visited. The bill and surrounding feather patches may be a precarious substrate for pollen adherence and therefore could have been a reason for the smaller pollen patches observed in *A. rufogularis*. This is especially important for honeyeaters as they typically perform a “bill whipping” behavior in which the bill is brushed along the perch after feeding^50^ which, if most of the pollen is on or near the bill as in *A. rufogularis*, could reduce the carried pollen load. In *Pu. albifrons* and *Pi. ornata* the pollen was placed on the crown (Fig. 1F, G), because the bill was shorter and narrower (Fig. 3) and was probed further inside the flower (Fig. 1F, G). The larger and flatter (more horizontally oriented) crown feathers could provide a more secure environment for the pollen grains to adhere to and therefore a large pollen patch could be created, and subsequently more pollen could be carried from the pollen donor flower to the pollen receiver flower. Previous work on honeyeater pollination has shown a similar trend, where pollen is typically deposited on the facial feathers of shorter billed honeyeaters and on the bill of longer billed honeyeaters^50^, suggesting that future research on pollen transfer by honeyeaters should investigate the concept of floral adaptive accuracy^7,8^ and where on the pollinator body the pollen is being placed. Two previous studies in sunbirds found results that echo what we found in *A. rufogularis*. Among several species of African avian pollinators, the morphologically specialized amethyst sunbird (*Chalcomitra amethystina*) is a worse pollinator of *Aloe ferox* plants than generalized, opportunistic nectar feeding birds such as weavers (*Ploceus ocularis*, *Ploceus cucullatus spilonotus*) and bulbuls (*Pycnonotus tricolor*)^47^. Similarly, in a Mediterranean avian pollinator community the morphologically specialized Palestine sunbird (*Cinnyris osea*) was a worse pollinator of *Anagyris foetida* plants than various species of warblers, bulbuls, and sparrows that visit plants to opportunistically consume nectar^61^. Like *A. rufogularis*, *Ch. amethystina* and *Ci. osea* have bills longer than the flowers they visited, while the generalist species have bills closer in size to, or shorter than these flowers. In these sunbird systems, the generalist birds had to stick their heads deeper into the flower and more pollen was deposited on their head feathers, which could then be carried to another flower^47,61^, while the sunbird either had some pollen deposited on the bill’s smoother surface or missed the reproductive structures all together.

An alternate, but not mutually exclusive, reason for the low pollen deposition by *A. rufogularis* could be their feeding efficiency. *A. rufogularis* had the highest feeding efficiency and therefore the shortest amount of time in contact with the flower, which could have resulted in the low rate of pollen deposition. The only interspecific difference in anther contact duration was between *Pu. albifrons* and *Pi. ornata* (Table 1), so it might not be that short feeding durations by *A. rufogularis* result in short duration of contact with the anthers, but rather with short duration of contact with the stigma; compounded with the differences in the location of the pollen patch across species, this could explain the low pollen deposition rates by *A. rufogularis*. While we did not find a correlation between bill-corolla matching and feeding efficiency (Table 2), there are other factors that could explain the interspecific differences observed (Fig. 1D, Table 1). *A. rufogularis* has a longer tongue than *Pi. ornata* and *Pu. albifrons*^62^ and a slower licking rate (Fig. 5). *A. rufogularis* may therefore need fewer licks than *Pu. albifrons* and *Pi. ornata* to extract the same amount of nectar because it can capture larger aliquots of nectar with each lick. While bill-corolla matching between the bill and the flower may not be important in determining honeyeater feeding efficiency at *E. maculata*, this variation in efficiency is congruent with differences seen in landscape-scale honeyeater foraging patterns. Larger bodied honeyeater species are known to visit more flowers at a faster rate before moving on to the next plant^51,63^, which could be due to increased feeding efficiency on a per-flower basis due to larger tongues, such as we observed in *A. rufogularis*.

Plant and landscape-level foraging patterns are important for determining bird contribution to plant fitness. When examining pollen deposition at various scales, we found that the interspecific patterns of pollen deposition on *E. maculata* stigmas changed from the scale of a single flower visit, a visit to an entire plant, or across the landscape (Fig. 6, Table 3). At the scale of the plant visit, *Pu. albifrons* was estimated to deposit a greater number of pollen grains per plant on average than *Pi. ornata* or *A. rufogularis* (Fig. 6A, Table 3); this pattern results from the fact that *Pu. albifrons* individuals had a higher mean pollen deposition per flower than the other two species (though not significantly different from *Pi. ornata*, Table 1, S1) with more high deposition visits (100+ grains deposited, Fig. 1A) and is not due to interspecific differences in the number of flowers probed per visit (Fig. 6C, Table 3). In contrast, at the landscape level *Pi. ornata* was estimated to deposit a greater number of pollen grains across all *E. maculata* plants observed via camera traps than *Pu. albifrons* or *A. rufogularis* (Fig. 6B, Table 3); this pattern results from the fact that *Pi. ornata* individuals were the most frequently observed species at *E. maculata* plants (Fig. 6D, Table 3). For self-incompatible plant species like *E. maculata*, the tendency for bird species to visit multiple flowers per plant could reduce the overall pollination efficiency and contribution to seed set of those species. For example, if larger honeyeaters with larger bills remove and deposit less pollen when moving between *E. maculata* flowers, such as *A. rufogularis* did in these experimental trials, and they visit the same number of flowers per plant as other honeyeater species, they may be largely transferring individual-own pollen to stigmas^64^. Existing evidence complicates this simple explanation, however. While not studied in *E. maculata* explicitly, past work on other Australian plants that are pollinated either in-full or in-part by honeyeaters has found high levels of seed paternal diversity. This finding suggests that there are likely other facets of honeyeater behavior in comparison to other pollinator groups (e.g., insects, non-flying mammals) beyond number of flowers probed per visit that affect the extent to which pollen is carried over within and between individual plants during foraging^65^. The distance traveled during foraging, the degree of plant fidelity during foraging, and the propensity to engage in aggressive interactions, which could dislodge pollen from feathers, mix pollen loads between interacting birds, or prevent certain pollinators from accessing flowers, could all be important.

Bill-corolla matching has been minimally considered as a force shaping honeyeater-plant interactions^50,54,55,64^. The current work demonstrates that bill-corolla matching is correlated with differences in pollen deposition across honeyeater species, demonstrating that the role of trait-matching in determining plant-bird interactions should be further investigated in this system. There are several limitations of the present work that should be investigated in further studies, the first being that fitness proxies were measured for both interaction partners rather than actual fitness. While pollen transfer is undoubtedly an important step in the process of pollination and per-visit pollen deposition is a common proxy of pollinator contribution to plant reproduction^66^, the relevance of it to ultimate measures of fitness such as seed set and seed genetic diversity vary depending on the breeding system of the plant species^66^. *E. maculata* is an outcrossing, self-incompatible species that has a high pollen/ovule ratio, meaning that many pollen grains are needed for sufficient fertilization of ovules^67^; this does not mean, however, that more pollen on the stigma causally equates to higher seed set and higher plant fitness, especially if birds probe multiple flowers per plant and deposit individual-own pollen. Future studies should quantify pollen limitation and seed set in *E. maculata* in the field to more directly determine the contribution of honeyeaters, and ideally individual honeyeater species, to plant fitness. Additional work should also be done examining differences in honeyeater pollination services between populations of *E. maculata*. The geographic mosaic theory of coevolution established that coevolution, for example that between plants and pollinators, occurs due to a mosaic of selection pressures in space and time between populations of interacting species^19–21^ and many studies support the geographic mosaic theory for plant-pollinator trait matching^25,33,68^. *E. maculata* is found throughout continental Australia, and while to our knowledge there are no other studies on honeyeater visitation to *E. maculata*, natural history accounts note that honeyeater species such as *Sogumel nigrium* (black honeyeater) and *Certhionyx variegatus* (pied honeyeater) visit the species in the more arid interior portions of the continent^69^. *S. nigrium* and *C. variegatus* are morphologically quite different than the three species examined here, they are smaller and have longer, thinner, more decurved bills^69^, and the ecological context between interior Australia and the temperate mallee ecosystem where this study was conducted are also notably different; conducting studies on these populations would allow for more convincing discussions of the presence or absence of trait matching-mediated coevolution between honeyeaters and *E. maculata*.

While much theoretical work on the evolution and maintenance of plant-pollinator relationships and networks has focused on invertebrates due to their tractability for manipulative and large-scale experiments, leveraging the phylogenetic power of the multiple independent origins of avian pollination could provide an ideal study system to address these questions and may produce very different answers, as there are fundamental differences between invertebrate and avian pollination systems^65^. For example, bird-pollinated plants have higher seed paternal diversity than insect-pollinated plants, which has been attributed to a number of life history and behavioral differences between birds and insects including higher mobility and aggression between individuals^65^. Building more bird-plant pollination networks that consider not only visitation rates but the contribution of each bird species to seed set and seed paternal diversity would allow for calculation of network-level metrics like connectance and specialization that are informed by the actual contribution of bird species to plant reproduction^65^. In insect pollination systems these metrics are known to relate to the robustness of plant-pollination interactions to extinction^70^, but they are usually only created with pollinator visitation data which does not accurately capture the contribution of each pollinator to plant fitness. Additionally, the increased paternal diversity offered by bird pollination may add additional robustness to bird-plant networks compared to insect networks making them less easily perturbed, even if bird-plant networks differ amongst themselves in metrics like conductance and specialization^54^. Considering the existing evidence documenting the ways in which native honeyeater-plant interactions are already being disrupted by invasive European honeybees^71,72^, conducting further work in the honeyeater pollination system is a necessary and time-sensitive endeavor.

## Methods

### Study site

This study was conducted at Gluepot Reserve in South Australia (−33.762375, 140.124265), which is in the Murray-Darling Depression Bioregion^73^ that spans much of southeastern South Australia as well as portions of New South Wales and Victoria. Gluepot is a roughly 54,500-hectare property and was pastoral land from 1877 until it was purchased by BirdLife Australia in 1997 and contains a mix of mallee woodland and casuarina woodland (specifically *Casuarina pauper*), with patches of open shrubland throughout due to the construction of dams (water impoundments for livestock) and the history of grazing. Mallee communities have experienced severe degradation and are cause for conservation concern in Australia^74^. The area encompassing Gluepot Reserve, along with Taylorville Station in the south, Calperum Station to the east and northeast, and Danggali Reserve to the north constitute one of the largest contiguous blocks of mallee woodland left in Australia^75^. Gluepot is typically dry, receiving 200-250 mm of rainfall per year.

### Camera trapping

There were no existing data on bird visitation to *E. maculata* flowers for Gluepot Reserve. To monitor bird visitation, we placed 16 cameras at *E. maculata* plants at four sites (four per site) across Gluepot; these sites were 9.2kilometers apart on average, with the closest two sites being 3.5 km apart and the furthest two sites being 10.5 km apart, and varied in size from roughly 0.5–1 hectare, with varying numbers of *E. maculata* plants per site. Because we were specifically interested in understanding which honeyeaters visited *E. maculata*, we placed our cameras at sites with high numbers of *E. maculata* plants and directing cameras towards plants that were in flower. Three of these sites were at dams surrounded by mallee, with minimal open shrubland. The final site did not have a dam and consisted of a small patch of open shrubland surrounded by mallee.

Cameras were deployed for a total of 2 weeks in September 2022 and were rotated to new plants within each site after the 1st week. Cameras were on 24 h per day and were motion activated, set to record 30-s video clips when triggered. This totaled 5376 h of camera deployment and 100 h of video, which were used to determine which honeyeater species were most frequently visiting *E. maculata* within Gluepot Reserve. All videos were watched by A.E. Hewes at 1x speed to determine the species and number of birds visiting the plant, as well as the number of flowers probed by each individual bird. The three species that were the most common were yellow plumed honeyeaters (*Pituloa ornata*), white fronted honeyeaters (*Purnella albifrons*) and spiny cheeked honeyeaters (*Acanthagenys rufogularis*) (Fig. S2), constituting >90% of visits. Because these three species were the most common (Supplementary Data 1) and exhibited a range of bill morphologies (Table S3), we focused on those species for the rest of the study.

### Bird handling and morphometry

We conducted mist netting from 0400–1200, the hours of peak bird activity in these habitats. We chose to mist net in areas of highest honeyeater density, which were also areas with large patches of *E. maculata* and the same areas we had previously placed camera traps. Nets were continuously monitored and birds removed immediately. Upon capture we used clear adhesive tape to clean the bird of existing visible pollen. Each bird was transferred from the point of capture to the field lab in an individual bird bag. After capture, bird morphometrics were measured in-hand before each bird was released into its 60 cm × 60 cm × 60 cm filming cage for acclimation. We measured body mass (g) using a Pesola spring scale and linear morphometrics using calipers. Wing chord (mm) was measured on a folded wing from the carpal joint to the tip of the longest primary feather, tarsus length (mm) was measured from intertarsal joint to the foot, bill length (mm) was measured from the feathers at the base of the bill to the tip of the bill, bill width (mm) was measured on the proximal side of the nares as the distance from one side of the bill to the other, and bill depth (mm) was measured on the proximal side of the nares as the height from the dorsal to ventral bill surface. The mean values ($\pm \,$SD) for all morphometrics for each species are given in Table S3.

Each bird was housed individually, and visual barriers were put between cages to minimize stress. No more than two birds were kept in captivity at the same time. Birds were given access to Wombaroo Lorikeet & Honeyeater Food (Wombaroo Passwell, Glen Osmond, South Australia) and a 20% sucrose solution (wt/wt) *ad libitum*. Birds were given several hours between capture and filming, during which time they were observed to ensure they were moving normally around the cage, preening, and feeding. To help birds acclimate to feeding on flowers while in captivity, we also gave birds access to several emasculated (stamens removed) *E. maculata* flowers filled with 20% sucrose (wt/wt). Any birds that were not feeding after several hours in captivity were released at the point of capture. All birds were caught, filmed, and released in the same day, and no birds were held in captivity for more than 8 h. Birds were marked on the crown with nail polish before release to prevent reuse if recaptured. We filmed 20 individuals: 9 *Pituloa ornata* (yellow-plumed honeyeater), 8 *Purnella albifrons* (white-fronted honeyeater), and 3 *Acanthagenys rufogularis* (spiny-cheeked honeyeater). All individuals were adults and sex was unknown (all species are sexually monomorphic). We have complied with all relevant ethical regulations for animal use. All work was done under University of Adelaide animal ethics approval S-2022-019 and animal trapping permit E27217-1 issued by the South Australian Department for Environment and Water, and permission from the Gluepot Management Committee was obtained prior to conducting field work.

### Collecting flowers

The genus *Eremophila* (family Scrophulariaceae) is endemic to Australia, where it is widespread and common in arid environments. Most species of *Eremophila*, including *E. maculata*, are perennial woody shrubs with densely arranged branches of hermaphroditic flowers^76,77^. Across *Eremophila* the flowers consist of 5 petals that vary in their degree of fusion, which tends to correspond with the primary pollen vector as species across the genus are differentially adapted to bird-based pollination (ornithophily) or insect-based pollination (entomophily)^77^. *E. maculata* is an ornithophilous species and this is reflected in its floral morphology; the petals are fused along their entire length except for the lower petal, which is only fused along roughly half its length and is reflexed back to form a lip, and the corolla varies in color but is most typically bright pink with dark pink spots (Fig. S3). *E. maculata* is self-incompatible and requires cross pollination from a conspecific to successfully set seed^67,76,77^. *E. maculata* is protandrous, the anthers dehisce before the stigma is completely grown to limit the number of self-pollen grains on the stigma^67,76^.

For feeding/pollen transfer trials we needed *E. maculata* flowers that had never been contacted by pollinators. We put pollinator exclusion bags on twelve *E. maculata* plants across three sites of the four sites we had previously placed camera traps, covering clusters of flowers that had not yet bloomed. Bags were checked daily to monitor blooming, particularly anther dehiscence. For flowers that were to be pollen recipients, anthers were clipped off using iris scissors before opening to reduce the chance of self-pollination. For flowers that were to be pollen donors, anthers were allowed to open but only flowers that had opened within the past 24 h were used in filming to reduce the chance of losing pollen to wind, rain, and other disturbance. The morning of each filming session, we went into the field and removed all viable pollen donor and recipient flowers from the bags on the *E. maculata* plants we were monitoring. Each flower was placed in a clean, covered plastic test tube and the plant ID and flower number were tracked. The tubes were kept in a test tube rack in a refrigerator (4 °C) until they were removed for photographing and filming. Flowers were used at random during filming and each flower was only used one time.

To ground truth whether our data on pollen deposition from experimental trials fell within a reasonable range, we collected flowers from the field to examine their pollen load. We collected stigmas from *E. maculata* flowers that were unlikely to be visited by pollinators due to the fact that the anthers had not opened and there was no nectar being produced (based on visual inspection of the flower). We also collected stigmas from visited flowers, which were producing nectar and had signs of visitation (some or all pollen removed from anthers). These flowers were collected at random from the *E. maculata* individuals we were observing, and their stigmas were prepared for examination under a microscope using the method described below. The pollen deposition data from the experimental trials fell between the range of pollen grains observed from stigmas of unvisited and visited flowers in the field, supporting the efficacy of the flower collection method (i.e., there was no contamination of stigmas prior to the experiment). The sample sizes for number of stigmas from unvisited and visited flowers, as well as for each honeyeater species from the experiments, are given in Table S4.

### Videography

Immediately before filming, flowers were photographed with a scale bar from the ventral and lateral perspective by a camera oriented perpendicular to the plane of interest. Floral morphometrics were quantified later in FIJI^78^ for calculation of bill-corolla matching (Fig. 2). The naturally occurring nectar was removed from each flower using 25 microliter microcapillary tubes and was replaced with 30 microliters of 27% sucrose solution (wt/wt) using a micropipette. The volume and concentration of sucrose solution employed in our experiments was determined by measuring nectar produced by *E. maculata* individuals at Gluepot using a Milwaukee MA871 digital brix refractometer allowing us to simulate the nectar available in an *E. maculata* flower as accurately as possible. Flowers were affixed to the wall of the cage perpendicular to the lateral view camera and were oriented such that they were parallel to the floor of the cage (in a standardized orientation commonly found in the wild *E. maculata* plants). The perch used to approach the flower and feed was set for each individual bird, adjusted during the post-capture acclimation period to ensure that the bird could reach the flower site comfortably, without showing signs of straining or difficulty while feeding.

Each bird participated in five two-part trials and the five trials were done in sequence. First, the bird was presented with a pollen donor *E. maculata* flower (flower that was collected within 24 h of dehiscence) and allowed to feed at that flower. The pollen donor flower was then removed from the cage and replaced with a pollen recipient flower (flower that had been emasculated before dehiscence and was stigma-only) and the bird was allowed to feed at that flower. The pollen recipient was subsequently removed from the cage and the process was repeated four more times, for a total of five donor and five recipient flowers per bird. Within pollen donor and pollen receiver groups, flowers were presented randomly with respect to plant ID to each bird (i.e., birds were not only presented with flowers from the same site in which they were captured); this was done to minimize the role of unmeasured site-level effects on flower morphology or traits relevant to pollen transfer (e.g., pollen load) on the parameters measured for this study. After each flower was removed from the cage it was dissected longitudinally and checked for remaining nectar. Any remaining nectar inside the flower was measured using a microcapillary tube. In 100% of trials the 30 microliters of nectar that was artificially placed into the anterior chamber of the corolla (Fig. S3) was completely consumed. In 10% of trials there was residual nectar on the surface of the nectary of the corolla (Fig. S3). Based on our floral dissections and observations of floral anatomy (Fig. S3) we know that access to the nectary of the corolla is physically obstructed by the filaments and ovary such that it is likely inaccessible to birds during feeding. We infer that the occasional appearance of residual nectar in the nectary represents floral nectar that was not fully removed via capillary tubes due to the constriction above the nectar (Fig. S3). Additionally, the 10% of trials where remaining nectar was observed were spread across 8 individuals from all 3 species (SC2, SC3, WF1, WF2, WF4, WF7, YP4, YP5), such that any effect would be unlikely to affect interspecific comparisons.

Each floral interaction was filmed at 800 frames per second from the dorsal and lateral perspectives using two Chronos 1.4 high-speed video cameras with Nikon AF Micro Nikkor 105 mm f/2.8 lenses. The high-speed videos were analyzed to measure proxies for pollen transfer and feeding efficiency (see below).

### Quantifying pollen acquisition

We measured two variables as proxies of pollen removal: the duration of contact between the bird and the anthers (*s*) and the area of the pollen patch left on the bird’s head (*mm*2). Both values were measured using the abovementioned high-speed videos, as the field of view for filming was sufficiently large to capture the entire contact between the bird’s head and the anthers. The structure of *E. maculata* anthers also makes contact easy to visualize, as the 4 filaments are elongated such that the anthers extend beyond the opening of the corolla and the anthers are reniform (kidney-shaped) creating a broad, flat surface on which to display pollen (Fig. S3). Duration of anther contact was measured using the video analysis software DLTdv8a^79^ to count the number of frames where the anthers were seen contacting the bird’s body (head and/or bill) and depositing pollen; frame number was converted to seconds dividing by the frame rate (800 fps) (Supplementary Data 2). The area of the pollen patch left on the bird’s head was measured using still images from dorsal-view videos in which the birds head was roughly perpendicular to the filming plane. Images were uploaded into FIJI^78^, known floral morphometrics were used to set the image scale in pixels/mm, and the draw polygon tool was used to outline the patch of deposited pollen and measure it in mm2 (Supplementary Data 2)_._

### Quantifying pollen deposition

Pollen deposition on the stigma of recipient flowers was measured using the flowers from the feeding/pollen transfer trials. After the bird’s visit, the flower was removed from the filming cage and the style was cut off and placed in a clean 1.2 mL microcentrifuge tube. Stigma samples were kept at room temperature until the end of the filming session for a given individual. Each stigma was flattened on a glass microscope slide and stained using basic fuchsin jelly^80^. The number of *E. maculata* pollen grains on the stigma were counted by viewing slides under an Olympus BX53 microscope (Supplementary Data 2). The pollen grains of *E. maculata* are similar in shape to many other genera of the tribe Myoporeae (family Scrophulariaceae) and they can be described as tri-colporate, isopolar, and radiosymmetrical^77^. To identify the pollen grains, we used Chinnock (2007) and the Australian Pollen and Spore Atlas. We did not find pollen grains of any other species in any of the microscope slides.

Because birds participated in five trials within a single day, they could have potentially accumulated some pollen on their body between trials. We chose not to recapture birds and physically remove pollen between trials with brushes or tape because it would have placed more stress on the birds, and because visiting multiple flowers in succession is more similar to what occurs in nature. Before conducting our statistical analyses, we examined whether there was an effect of trial on pollen deposition, and we found no such effect (see “Modeling pollen transfer and feeding efficiency” section for details, Fig. S4).

### Quantifying feeding efficiency

Total time spent feeding was measured, along with feeding efficiency in microliters/second, as 30 microliters divided by the duration of the feeding bout in seconds, which was measured from the high-speed videos using the video analysis software DLTdv8a^79^. We used forward hyoid movement (visible in the throat, indicative of forward tongue movement) as the marker of the beginning of a feeding bout and used the hyoid coming to rest in the throat, just before the bill was pulled out of the flower, as the marker of the end of a feeding bout; frame number was converted to seconds by dividing by the frame rate (800 fps) (Supplementary Data 3). Finally, to better characterize differences in feeding efficiency across the three species we also measured licking rate. The number of licks was counted by counting each protrusion/retraction cycle of the tongue, or each cycle of hyoid movement in cases were the tongue was obscured by the floral corolla. The number of licks was counted and divided by the total feeding bout time to get a measurement of licks/second (Supplementary Data 3).

### Quantifying bill-corolla matching using PCA

We define bill-corolla matching as the ratio between bird and floral linear morphometrics, for example bill length divided by corolla length. The definition of each floral measurement and the corresponding bill-corolla matching metric are given in Fig. 2. We focused on metrics that were ratios of width and lengths, as those metrics have been found to be important in previous work on bird-plant feeding interactions^50^, and tarsus length was used in analyses to account for body size^81^. In the raw data, a value of 1 indicates the two structures have the same value for a given bill-corolla matching metric, a value <1 indicates that the floral value is greater than the bird value (e.g., corolla is longer than bill), and a value >1 indicates that the bird value is greater than the floral value (e.g., bill is longer than corolla) (Fig. 2B–F) (Supplementary Data 4).

Because we had a number of bill-corolla matching metrics to investigate (Fig. 2), we used a principal component analysis (PCA) to reduce the number of dimensions of the morphological data. We ran one PCA for bird interactions with the pollen donor flowers and a second PCA for bird interactions with the pollen receiving flowers using the prcomp() function in the stats package v 4.2.3^82^ in R v 4.2.3^82^. In both PCAs the data were centered and scaled as part of the analysis. We distinguish between these PC scores by noting “pollen donor PC1” and “pollen receiver PC1”, “pollen donor PC2” and “pollen receiver PC2”, etc (Supplementary Data 4). In the models where bill-corolla matching is investigated (Table 2), we used the absolute value of all PC scores (i.e., the magnitude of the score only, not the sign) as it greatly improved the fit of the models and our ability to interpret the results. A PC score of 0 indicates the closest bill-corolla match. The further away from zero the PC score, the more mismatched the bill and corolla are.

### Statistics and reproducibility

#### Reproducibility

We collected data on 20 individual birds from 3 species: 9 *Pituloa ornata* (yellow-plumed honeyeater), 8 *Purnella albifrons* (white-fronted honeyeater), and 3 *Acanthagenys rufogularis* (spiny-cheeked honeyeater). The complete dataset of feeding/pollen transfer interactions contains 100 bird-flower trials composed of 100 pollen donor visits and 100 pollen recipient visits, totaling 200 individual flower visits.

#### Modeling pollen transfer and feeding efficiency

All statistical analyses were conducted in R v 4.2.3^82^ with alpha = 0.05. We began by determining whether there was an effect of trial number on the amount of pollen deposition. Due to the unbalanced number of observations across species, we used the Anova() function with a type III sum of squares from the car package v 3.1-2^83^ to determine whether there was a significant effect of trial number, species, or the interaction between trial number and species on the number of pollen grains deposited on *E. maculata* stigmas during experimental trials. As neither trial number nor trial*species were significant (Fig. S4), we did not incorporate trial as a predictor in any of the following models. We then used generalized linear mixed effects models (GLMMs) to determine whether honeyeater species differ in metrics of pollen transfer: pollen deposition on stigmas (Mod1), anther contact duration (Mod2), or the area of the pollen patch (Mod3), or in metrics of feeding efficiency: feeding duration (Mod4) or microliters of nectar consumed per second (Mod5). We used GLMMs to answer these questions because we did not expect the data to fit all assumptions of a standard linear model, namely, independence of residuals, as each bird was measured multiple times, and a Gaussian error distribution, as our data was either counts (e.g., number of pollen grains deposited on the stigma) or positive and continuous, with or without zeros (e.g., feeding efficiency). All models were run using the glmmTMB() function in the glmmTMB package v 1.1.7^84^.

In Mod1-Mod5 the fixed effect of interest was species and individual bird ID was included as a random effect. We were not interested in intra-individual differences per se but needed to account for them statistically as each bird participated in multiple trials. Pollen deposition on stigmas was modeled using a negative binomial distribution with a log link, due to the fact that the response variable was counts with large variance relative to the mean. Duration of anther contact and area of pollen patch were modeled with a Tweedie distribution with a log link because our response variables were continuous, positive and included zeros, making other distributions such as Gaussian or Gamma inaccurate fits. Finally, feeding duration and feeding efficiency (volumetric intake rate) was modeled using a gamma distribution with a log link, as our response variables were continuous, positive, and non-zero.

The fit of all models was checked with model validation operations in the DHARMa package v 0.4.6^85^. In cases where zero inflation affected model fit, a zero-inflation parameter by species was incorporated into the model (Mod2 and Mod3). For all models, the Anova() function in the car package v 3.1-2^83^ was used to extract *p* values for the fixed effect of “species” and the function contrast() from the emmeans package v 1.8.5^86^ was used to extract pairwise *p* values across levels of the fixed effect. The cohens_d() function from the rstatix package v 0.7.2^87^ to was used to calculate the pairwise effect sizes across levels of the fixed effect. Marginal and conditional R2 were estimated using the r.squaredGLMM() function from the MuMIn package v 1.47.5^88^.

#### Modeling trait matching and the correlation with pollen transfer and feeding efficiency

Having established that honeyeater species differ in bill-corolla matching using PCA and in metrics of pollen transfer and feeding efficiency in Mod1-Mod5, we then used GLMMs to determine whether bill-corolla matching is correlated with area of the pollen patch (Mod6), pollen deposition on the stigma (Mod7), or microliters of nectar consumed per second (Mod8).

In Mod6-8 the explanatory variable of interest was trait matching, but we needed to account for multiple measurements per individual and for the fact that individuals belong to distinct species, so we included the random effects of individual nested within species. Mod6 examines the relationship between the area of the pollen patch the birds had after visits to pollen donor flowers and their trait matching with those flowers. We used pollen donor PC1 and pollen donor PC2 as predictors to quantify trait matching, as the first two PCs explain over 85% of the variance. Mod7 examines the relationship between the number of pollen grains birds deposited on the stigma of pollen receiver flowers and their trait matching with those flowers, as well as the size of the pollen patch they had removed at their previous pollen donor flower visit; in this model we used pollen receiver PC1 and pollen receiver PC2 as predictors to quantify trait matching, as the first two PCs explain over 85% of the variance. Mod8 examines the relationship between birds’ volumetric uptake rate of nectar during visits to both pollen donor and pollen receiver flowers and their trait matching with those flowers; we used PC1 and PC2 from pollen donor and pollen receiver PCs. The fit of all models was checked with model validation operations in the DHARMa package v 0.4.6^85^. The Anova() function in the car package v 3.1-2^83^ was used to extract *p* values for the fixed effects. Marginal and conditional R2 were estimated using the r.squaredGLMM() function from the MuMIn package v 1.47.5^88^.

### Scaling up to the pollination landscape

The experiments described above quantify pollen deposition in a single flower visit, but our camera trap data showed that these three species have different visitation frequencies to *E. maculata* plants in the field. We combined our data on visitation frequency and number of flowers probed per plant from camera traps with data on pollen deposition from pollen transfer experiments to determine how these three species differ in their estimated pollen deposition at the scale of an entire plant visit and visiting multiple plants across the landscape. For all visits by *A. rufogularis, Pi. ornata*, and *Pu. albifrons* recorded on camera traps, we used a permutation approach where a random value for pollen deposition, sampling from the values measured in pollen transfer experiments, was assigned to a given camera trap observation. Pollen deposition values were only sampled within each species and values were sampled with replacement, as there are more camera trap observations than pollen transfer trials per species. Once pollen deposition values were assigned to each camera trap observation, the number of pollen grains deposited during the entire plant visit was determined by multiplying the number of pollen grains assigned to that observation by the number of flowers probed during that observation, as counted by A.E.Hewes when examining camera trap videos. The mean number of pollen grains deposited per visit per species was then calculated for that permutation run (Fig. 6A). The number of pollen grains deposited across the landscape was determined by summing all per-visit pollen deposition estimates per species for each permutation run (Fig. 6B). We ran 100 permutations to generate a distribution of pollen deposition estimates for each species (Supplementary Data 5).

Due to the unbalanced number of observations across species, we used the Anova() function with a type III sum of squares from the car package v 3.1-2^83^ to determine whether there were interspecific differences in average number of pollen grains deposited per visit and total pollen grains deposited across the landscape, using the data generated from this permutation approach, as well as the number of flowers probed per visit from our raw camera trap data. We then used the pairs() function from the emmeans package v 1.8.5^86^ to conduct a TukeyHSD post-hoc test to calculate *p* values for each pairwise species comparison and the cohens_d() function from the rstatix package v 0.7.2^87^ to calculate the effect sizes of each pairwise species comparison (Table 3).

### Reporting summary

Further information on research design is available in the Nature Portfolio Reporting Summary linked to this article.

## Supplementary information

Supplemental Information

Description of Additional Supplementary Files

Supplementary Data 1

Supplementary Data 2

Supplementary Data 3

Supplementary Data 4

Supplementary Data 5

Reporting Summary

## Data Availability

All source data underlying the analyses, graphs, and charts presented in this article are in the Supplementary Data.

## Code availability

Code is available upon request to the corresponding author.
