# Supplementary material for: Plant-pollinator trait matching affects pollen transfer but not feeding efficiency of Australian honeyeaters (Aves, Meliphagidae)
Source: Commun Biol. 2025 Mar 1;8:339. doi: 10.1038/s42003-025-07693-w (PMC11871056; doi:10.1038/s42003-025-07693-w)
Supplement: Supplementary file 1 — Supplemental Information [file 42003_2025_7693_MOESM1_ESM.pdf]

SUPPLEMENTAL INFORMATION

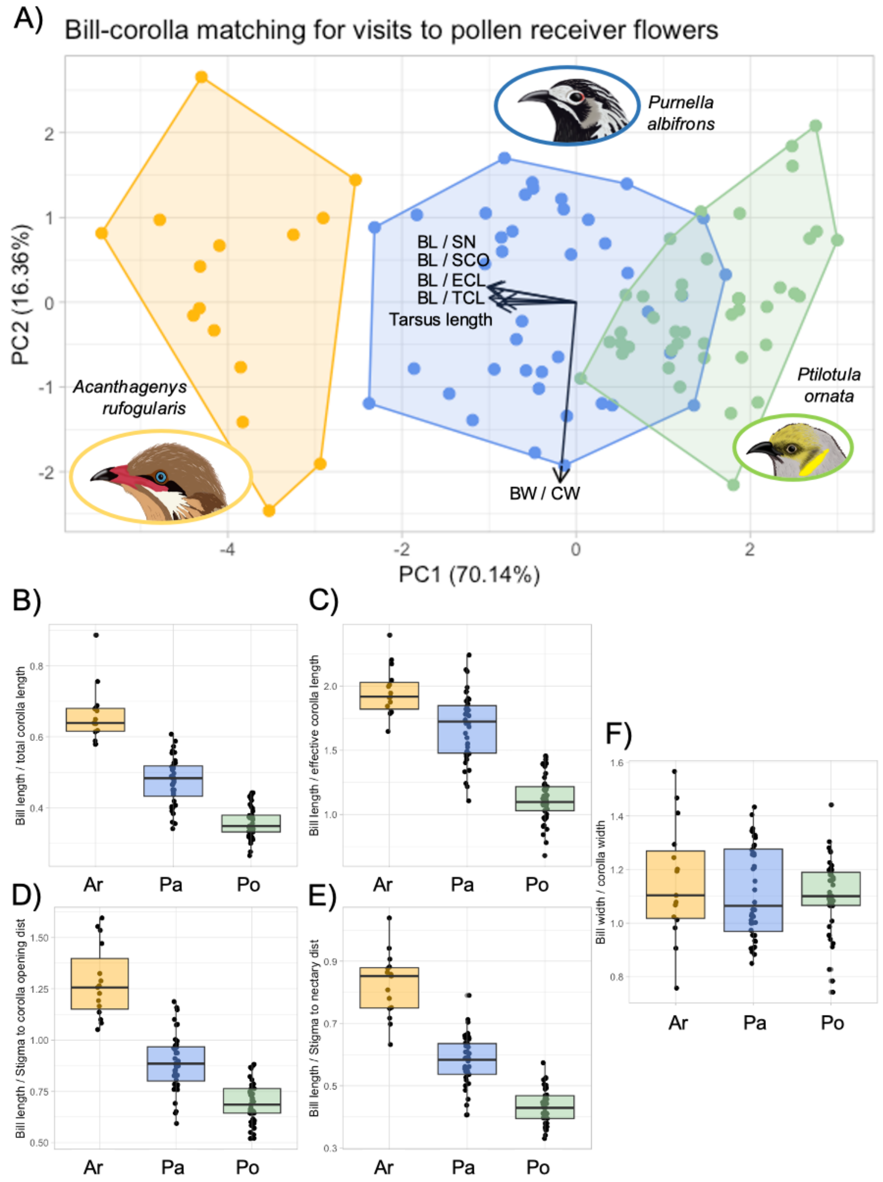

Figure S1. A) PCA of bill-corolla matching metrics measured from honeyeater visits to pollen receiver *Eremophila maculata* flowers. The loadings of each bill-corolla matching variable are illustrated with arrows on both plots. B-F) Raw data of bill-corolla matching metrics showing the same trends visualized in the PCA. In A abbreviations are as follows: BL / TCL = bill length / total corolla length, BL / ECL = bill length / effective corolla length, BW / CW = bill width / corolla width, BL / ACO = bill length / distance from anthers to corolla opening, BL / AN = bill length / distance from anthers to nectary, BL / SCO = bill length / distance from stigma to corolla opening BL / SN = bill length / distance from stigma to nectary. See Figure 2 for descriptions of all bill-corolla matching variables, Table S2 for PCA loadings, and Figure 3A for PCA of bill-corolla matching metrics measured from honeyeater visits to pollen donor flowers. Bird illustrations (to scale) are by Kindall Murie.

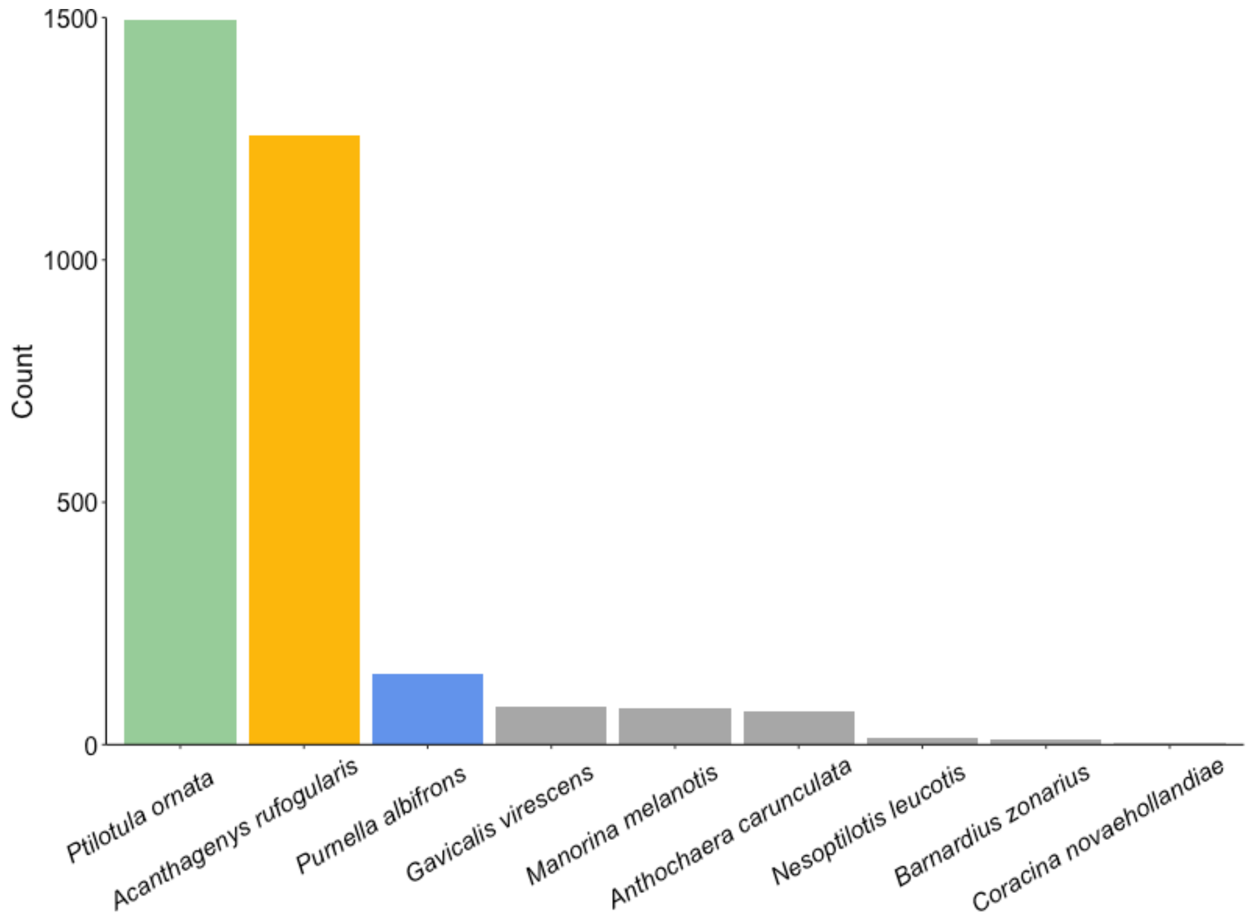

Figure S2. Visitation of all bird species to *Eremophila maculata* plants recorded on camera traps in the field.

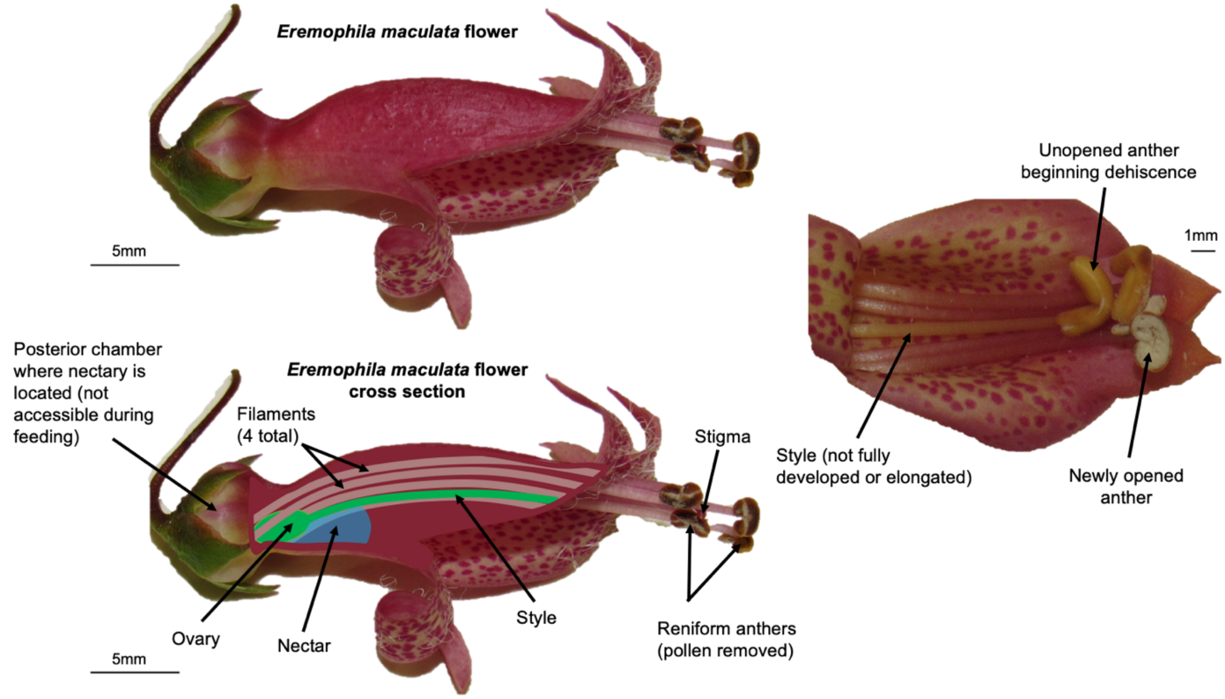

Figure S3. Relevant anatomical features of *Eremophila maculata* flowers. Blue shading shows where naturally occurring nectar pools for consumption by pollinators, after being produced by secretary pores of nectary (Chinnock 2007b) in the posterior chamber behind the ovary. The nectar shown would have been removed via capillary tube and replaced with a sucrose solution before being used in feeding/pollen transfer experiments.

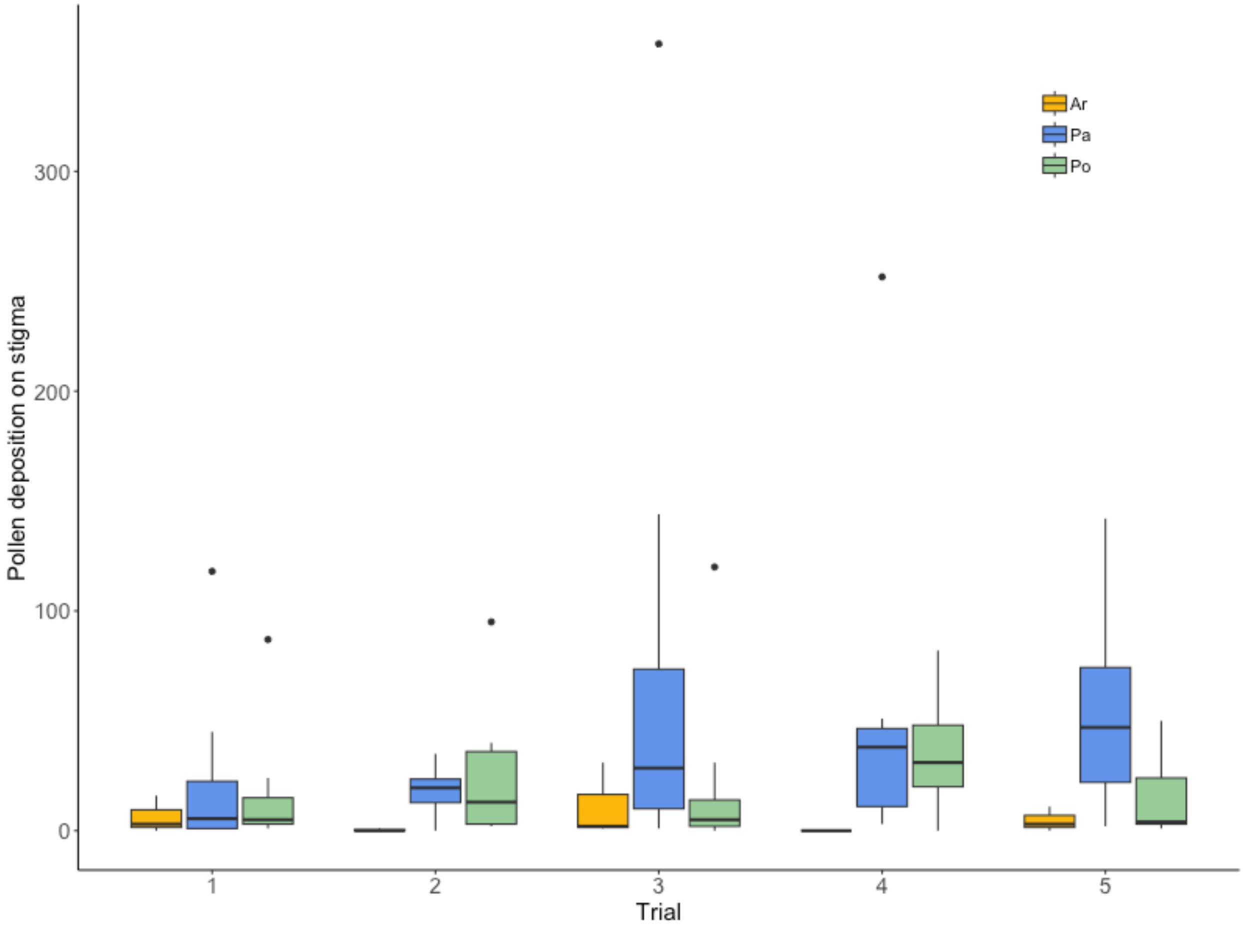

Figure S4. Pollen deposition on *Eremophila maculata* stigmas for each trial. There was no significant relationship between pollen deposition and trial number (F statistic = 0.581, p-value = 0.677), or between pollen deposition and the interaction of species and trial number (F statistic = 0.656, p-value = 0.729). Ar = *Acanthagenys rufogularis,* Pa = *Purnella albifrons*, and Po = *Ptilotula ornata*.

Table S1. Pollen transfer and feeding metrics across species (mean ± SE).

Species	Pollen deposition on stigma (count)	Anther contact duration (s)	Pollen patch area (mm^2)	Feeding duration (s)	Feeding efficiency (µL/s)	Licking rate (licks/s)
Acanthagenys rufogularis	4.5 ± 2.2	2.1 ± 0.38	8.8 ± 2.2	2.8 ± 0.22	13.4 ± 1.3	12.92 ± 0.18
Pituloa ornata	22.5 ± 4.2	4.4 ± 0.46	27.4 ± 2.7	5.6 ± 0.31	7.0 ± 0.43	14.95 ± 0.35
Purnella albifrons	46.7 ± 11.6	1.8 ± 0.31	21.6 ± 3.8	5.3 ± 0.33	7.3 ± 0.47	15.92 ± 0.18

Table S2. Results of PCAs for pollen donor and pollen receiver flower interactions. The loadings for each bill-corolla matching trait on each principle component are shown in rows, while the variance explained by each principle component is shown at the top of each column. The abbreviations for the bill-corolla matching variables in each row are defined in Figure 2.

	**Principle component (proportion of variance)**	**PC1 (71.7%)**	**PC2 (15.2%)**	**PC3 (7.8%)**	**PC4 (4.0%)**	**PC5 (1.1%)**	**PC6 (0.17%)**
**Pollen donor PCA**	BL / TCL	-0.4654	0.1175	0.0687	-0.1184	0.8650	0.0517
	BL / ECL	-0.4068	0.3027	-0.4725	-0.6194	-0.3184	0.1859
	BW / CW	-0.2035	-0.9210	-0.3168	-0.0945	0.0288	-0.0162
	BL / ACO	-0.4544	0.0442	-0.0766	0.6483	-0.1900	0.5739
	BL / AN	-0.4701	0.1199	-0.1117	0.3022	-0.1715	-0.7946
	Tarsus length	-0.3860	-0.1733	0.8083	-0.2858	-0.2900	0.0425
	**Principle component (proportion of variance)**	**PC1 (70.1%)**	**PC2 (16.4%)**	**PC3 (8.1%)**	**PC4 (3.6%)**	**PC5 (1.6%)**	**PC6 (0.26%)**
**Pollen receiver PCA**	BL / TCL	-0.4659	0.0243	-0.1389	0.1605	0.8582	0.0272
	BL / ECL	-0.4241	-0.0153	-0.6006	0.4888	-0.4112	-0.2262
	BW / CW	-0.0860	-0.9926	0.0119	-0.0826	-0.0018	0.0214
	BL / SCO	-0.4606	0.0830	0.0762	-0.6551	-0.0992	-0.5798
	BL / SN	-0.4765	0.0841	-0.0544	-0.3152	-0.2356	0.7798
	Tarsus length	-0.3956	0.0055	0.7817	0.4473	-0.1702	-0.0584

Table S3. Honeyeater morphometrics. Tongue morphometrics are from Paton and Collins (1989), all others are average values ± SD measured from individual birds used in the present study.

Species (n)	Bill length (mm)	Bill width (mm)	Bill depth (mm)	Wing chord (mm)	Tarsus length (mm)	Capture mass (g)	Tongue length (mm)	Tongue width (mm)	Tongue depth (mm)
Acanthagenys rufogularis (3)	19.93 ± 2.11	5.55 ± 0.96	5.62 ± 0.54	110 ± 4.36	25.11 ± 0.19	45 ± 2.0	24.7	0.88	0.78
Purnella albifrons (8)	13.36 ± 1.32	4.52± 0.36	4.29 ± 0.37	75 ± 4	17.76 ± 1.27	15.11 ± 1.89	21.6	0.83	0.59
Ptilotula ornata (9)	10.59 ± 1.03	4.81 ± 0.15	4.24 ± 0.20	79.9 ± 5.26	17.56 ± 0.92	16.91 ± 2.0	16.7	0.93	0.73

Table S4. Sample size information for pollen deposition analyses

**Treatment**	**Number of individuals (plant or bird)**	**Total number of stigmas sampled**
Acanthagenys rufogularis	3	15
Purnella albifrons	8	40
Ptilotula ornata	9	45
Unpollinated baseline (anthers unopened, unbagged)	8	26
Pollinated baseline (anthers opened, unbagged)	15	61
