# Supplementary material for: Plant-pollinator trait matching affects pollen transfer but not feeding efficiency of Australian honeyeaters (Aves, Meliphagidae)
Source: Commun Biol. 2025 Mar 1;8:339. doi: 10.1038/s42003-025-07693-w (PMC11871056; doi:10.1038/s42003-025-07693-w)
Supplement: Supplementary file 2 — Description of Additional Supplementary Files [file 42003_2025_7693_MOESM2_ESM.pdf]

Description of Additional Supplementary Files

File name: Supplementary Data 1

Description: Bird visitation to Eremophila maculata source data

File name: Supplementary Data 2

Description: Pollen transfer source data (area of pollen patch, another contact duration, pollen deposition)

File name: Supplementary Data 3

Description: Feeding measurements source data (feeding duration, feeding efficiency, lick frequency)

File name: Supplementary Data 4

Description: Bill-corolla matching metrics source data and PCA scores

File name: Supplementary Data 5

Description: Landscape-scale permutations of pollen deposition
