# Supplementary material for: Plant-pollinator trait matching affects pollen transfer but not feeding efficiency of Australian honeyeaters (Aves, Meliphagidae)
Source: Commun Biol. 2025 Mar 1;8:339. doi: 10.1038/s42003-025-07693-w (PMC11871056; doi:10.1038/s42003-025-07693-w)
Supplement: Supplementary file 8 — Reporting Summary [file 42003_2025_7693_MOESM8_ESM.pdf]

nature portfolio

Corresponding author(s): Amanda E. Hewes

Last updated by author(s): 1/10/2025

Reporting Summary

Nature Portfolio wishes to improve the reproducibility of the work that we publish. This form provides structure for consistency and transparency in reporting. For further information on Nature Portfolio policies, see our Editorial Policies and the Editorial Policy Checklist.

Statistics

For all statistical analyses, confirm that the following items are present in the figure legend, table legend, main text, or Methods section.

n/a	Confirmed	
☐	☒	The exact sample size (*n*) for each experimental group/condition, given as a discrete number and unit of measurement
☐	☒	A statement on whether measurements were taken from distinct samples or whether the same sample was measured repeatedly
☐	☒	The statistical test(s) used AND whether they are one- or two-sided<br>*Only common tests should be described solely by name; describe more complex techniques in the Methods section.*
☐	☒	A description of all covariates tested
☐	☒	A description of any assumptions or corrections, such as tests of normality and adjustment for multiple comparisons
☐	☒	A full description of the statistical parameters including central tendency (e.g. means) or other basic estimates (e.g. regression coefficient) AND variation (e.g. standard deviation) or associated estimates of uncertainty (e.g. confidence intervals)
☐	☒	For null hypothesis testing, the test statistic (e.g. *F*, *t*, *r*) with confidence intervals, effect sizes, degrees of freedom and *P* value noted<br>*Give P values as exact values whenever suitable.*
☒	☐	For Bayesian analysis, information on the choice of priors and Markov chain Monte Carlo settings
☒	☐	For hierarchical and complex designs, identification of the appropriate level for tests and full reporting of outcomes
☐	☒	Estimates of effect sizes (e.g. Cohen's *d*, Pearson's *r*), indicating how they were calculated

Our web collection on statistics for biologists contains articles on many of the points above.

Software and code

Policy information about availability of computer code

Data collection	DLTdv8 and FIJI were the only software programs used for data collection. Both of these software programs are free and open source, and their relevant citations are in the main text.
Data analysis	R v 4.2.3 was used for all statistical analyses. Citation provided in main text.

For manuscripts utilizing custom algorithms or software that are central to the research but not yet described in published literature, software must be made available to editors and reviewers. We strongly encourage code deposition in a community repository (e.g. GitHub). See the Nature Portfolio guidelines for submitting code & software for further information.

Data

Policy information about availability of data

All manuscripts must include a data availability statement. This statement should provide the following information, where applicable:

- Accession codes, unique identifiers, or web links for publicly available datasets
- A description of any restrictions on data availability
- For clinical datasets or third party data, please ensure that the statement adheres to our policy

All source data underlying the analyses, graphs and charts presented in this article are in the Supplementary Data.

Human research participants

Policy information about studies involving human research participants and Sex and Gender in Research.

Reporting on sex and gender	Not applicable
Population characteristics	Not applicable
Recruitment	Not applicable
Ethics oversight	Not applicable

Note that full information on the approval of the study protocol must also be provided in the manuscript.

Field-specific reporting

Please select the one below that is the best fit for your research. If you are not sure, read the appropriate sections before making your selection.

☐ Life sciences ☐ Behavioural & social sciences ☒ Ecological, evolutionary & environmental sciences

For a reference copy of the document with all sections, see nature.com/documents/nr-reporting-summary-flat.pdf

Ecological, evolutionary & environmental sciences study design

All studies must disclose on these points even when the disclosure is negative.

Study description	This study examines how pollen transfer efficiency and feeding efficiency vary across three species of honeyeater (Aves, Meliphagidae), and whether bill-flower matching plays a role in determining interspecific differences in feeding and pollen transfer efficiency. We use manipulative techniques in the field in South Australia to recreate bill-flower pollination interactions in a controlled by real-to-life way.
Research sample	We measured 20 birds total for this study: 9 Pituloa ornata (yellow-plumed honeyeater), 8 Purnella albifrons (white-fronted honeyeater), and 3 Acanthagenys rufogularis (spiny-cheeked honeyeater). Each bird participated in 5 trials where data was collected, which we account for in the statistical analyses.
Sampling strategy	Sample size was dependent on the number of birds in the field and the rate at which we were able to catch them via mist netting. Each bird participated in 5 trials, which we account for in the statistical analyses.
Data collection	The data collection procedure involved capturing wild birds via mist netting, collecting newly bloomed flowers from the field, and recreating bird-flower pollination interactions in a field lab. Each bird was presented with a pollen donor flower followed by a pollen receiver flower, and we measured the removal and deposition of pollen between flowers and the rate at which the bird consumed the nectar from each flower using high-speed videos. We also collected data on bird and flower morphometrics to calculate the degree of bill-flower matching in parameters like length and width. Each bird participated in 5 trials of pollen donor flower+pollen receiver flower and each flower was only used in 1 trial. Data was collected by AEH and TJM.
Timing and spatial scale	Data was collected from September - November 2022, as it was the time of peak bloom for our focal plant species, and we collected data using flowers and birds from Gluepot Reserve, which is a roughly 54,500 hectare reserve.
Data exclusions	No data were excluded from analyses.
Reproducibility	The methods provided are in depth to ensure reproducibility. Similar studies could be conducted following the same methodology.
Randomization	Birds were processed in the order in which they were caught in the mist net. Birds were not held in captivity for more than 1 day, so we worked with birds in the order in which they were caught. In the experimental procedure, birds were presented with a flower from a random plant individual to minimize the role of unmeasured site-level effects on flower morphology or traits relevant to pollen transfer (e.g., pollen load) on the parameters measured for this study. Other than that, there were no parameters to randomize.
Blinding	Because we are specifically interested in differences between honeyeater species, blinding was not relevant for this work.

Did the study involve field work? ☒ Yes ☐ No

Field work, collection and transport

Field conditions	Field work was conducted on Gluepot Reserve in South Australia. At the time of the year we were collecting data, the temperature ranged from 20-35 degrees Celsius and the rainfall can be extremely variable, but the climate is generally dry. Gluepot is a mallee ecosystem, meaning it is dominated by low lying Eucalyptus trees.
Location	This study was conducted at Gluepot Reserve in South Australia (-33.762375, 140.124265), which is a roughly 54,500 hectare property
Access & import/export	There was no import-export. All data was collected at the reserve and all birds were released after filming.
Disturbance	No disturbance was caused. We received permission from the Gluepot Management Committee before conducting our field work.

Reporting for specific materials, systems and methods

We require information from authors about some types of materials, experimental systems and methods used in many studies. Here, indicate whether each material, system or method listed is relevant to your study. If you are not sure if a list item applies to your research, read the appropriate section before selecting a response.

Materials & experimental systems

n/a	Involved in the study	
☒	☐	Antibodies
☒	☐	Eukaryotic cell lines
☒	☐	Palaeontology and archaeology
☐	☒	Animals and other organisms
☒	☐	Clinical data
☒	☐	Dual use research of concern

Methods

n/a	Involved in the study	
☒	☐	ChIP-seq
☒	☐	Flow cytometry
☒	☐	MRI-based neuroimaging

Animals and other research organisms

Policy information about studies involving animals; ARRIVE guidelines recommended for reporting animal research, and Sex and Gender in Research

Laboratory animals	This study did not involve laboratory animals
Wild animals	Bird were observed with camera traps to understand which species visited our plant of interest. Birds were then captured using mist nets and were not held in captivity for more than 1 day. Individuals caught were adults, but sex could not be determined because the species are monomorphic (no morphological differences between males and females). Individuals were released at the point of capture after filming and were marked on the crown with nail polish to prevent recapture.
Reporting on sex	Sex could not be determined for the birds we worked with because the species are monomorphic (no morphological differences between males and females). This is mentioned in the Methods section of the text.
Field-collected samples	Birds were not held in captivity for more than 1 day. No more than 2 birds were kept in captivity and any time and visual barricades were set up between cages to limit stress. Birds were housed in individual cages and given free access to sugar water and Wombaroo nectarivorous bird food mix. The field lab was open to the environment, so temperature and photoperiod were all kept at natural conditions. If any individuals did not begin exhibiting normal behaviors (preening, feeding, moving around the cage) within a set time after capture, they were released. No birds were euthanized for this study.
Ethics oversight	All work was conducted under University of Adelaide animal ethics approval S-2022-019 and animal trapping permit E27217-1 issued by the South Australian Department for Environment and Water. This information is also in the main text. While not an animal ethics body, we also received permission from the Gluepot Management Committee before conducting our field work.

Note that full information on the approval of the study protocol must also be provided in the manuscript.
